# Low-Fat by Formulation, Not by Benefit: A Purified Low-Fat Diet Is Associated with Gut–Liver–Kidney Alterations in Rats

**DOI:** 10.3390/nu18213718

**Published:** 2026-11-09

**Authors:** Pedro Vieira, Gonçalo Ferreira, Filipa Carvalho, Carolina Ferreira, André Alves, Pedro Gomes, Tânia Martins-Marques, Tânia Ribeiro, Pedro Castro, Mónica Zuzarte, Henrique Girão, Manuela Pintado, Lígia Salgueiro, Flávio Reis, Sofia Viana

**Affiliations:** 1 Institute of Pharmacology & Experimental Therapeutics Faculty of Medicine, University of Coimbra, 3000-548 Coimbra, Portugal; pedromdvieira96@gmail.com (P.V.); goncalocrferreira@gmail.com (G.F.); filipa.2002@sapo.pt (F.C.); f202carolina@gmail.com (C.F.); alves.andrefb@gmail.com (A.A.); pgomes@med.up.pt (P.G.); tania.m.marques@fmed.uc.pt (T.M.-M.); pedro.aam.castro@gmail.com (P.C.); freis@fmed.uc.pt (F.R.); 2 Coimbra Institute for Clinical and Biomedical Research (iCBR), Faculty of Medicine, University of Coimbra, 3000-548 Coimbra, Portugal; mzuzarte@uc.pt (M.Z.); hmgirao@fmed.uc.pt (H.G.); 3 CIBB—Center for Innovative Biomedicine and Biotechnology, University of Coimbra, 3004-504 Coimbra, Portugal; 4 CACC—Clinical Academic Center of Coimbra, 3004-531 Coimbra, Portugal; 5 CBQF—Centro de Biotecnologia e Química Fina—Laboratório Associado, Escola Superior de Biotecnologia, Universidade Católica Portuguesa, Rua Diogo Botelho 1327, 4169-005 Porto, Portugal; tribeiro@ucp.pt (T.R.); mpintado@ucp.pt (M.P.); 6 Nephrology Department, Unidade Local de Saúde de Coimbra, 3000-076 Coimbra, Portugal; 7 FFUC—Faculty of Pharmacy, University of Coimbra, 3000-548 Coimbra, Portugal; ligia@ff.uc.pt; 8 CERES—Chemical Engineering and Renewable Resources for Sustainability, Department of Chemical Engineering, University of Coimbra, 3030-790 Coimbra, Portugal

**Keywords:** low-fat, carbohydrate quality, dietary fiber, free sugars, short-chain fatty acids (SCFAs), uremic toxins, gut-liver-kidney axis

## Abstract

**Background/Objectives:** “Low-fat” claims are defined by a fat threshold, with no requirements regarding the nutrients replacing fat. Reformulation often relies on refined starches and free sugars at the expense of dietary fiber. Thus, a low-fat (LF) product is not necessarily of good carbohydrate quality, a dimension that macronutrient-based classification fails to capture. Whether LF-derived poor carbohydrate quality is associated with organ-specific alterations across the gut–liver–kidney axis remains unknown. **Methods:** Male Wistar rats were fed for 24 weeks a purified LF diet, its ingredient-matched purified high-fat, high-sucrose counterpart (HF), or a whole-grain Chow reference of similar energy density. Carbohydrate quality was assessed using the carbohydrate-to-dietary fiber-to-free sugars ratio. Body weight and adiposity, glucose homeostasis, gut fermentation, and hepatic and renal functional and structural parameters were evaluated. **Results:** Only Chow met the 10:1:1 carbohydrate quality criterion. At a caloric intake that did not exceed that of Chow, LF feeding was associated with increased body weight, adipocyte hypertrophy, impaired glucose tolerance and postprandial hyperinsulinemia. Fecal short-chain fatty acids (SCFAs) and branched SCFAs declined, consistent with a reduction in colonic fermentative output. Chronic LF exposure was additionally associated with hepatic and renal lipid accumulation, normoglycemic glycosuria, and podocyte structural alterations. **Conclusions:** A purified low-fat diet was associated with the same spectrum of gut, liver and kidney alterations as its high-fat counterpart, without excess caloric intake. These findings support an important role for nutrient sources and carbohydrate quality in shaping metabolic health beyond dietary fat content or caloric load alone.

## 1. Introduction

Dietary fat restriction has long been a cornerstone of nutritional guidance for the prevention and management of cardiometabolic disease. Current international recommendations advocate that total fat intake should not exceed approximately 30% of total daily energy intake, particularly emphasizing the reduction of saturated fatty acids, as adherence to low-fat dietary patterns has been associated with improvements in cardiovascular risk factors, lipid profile and overall metabolic health [1]. These recommendations have profoundly influenced both consumer behavior and food manufacturing practices, leading to the widespread commercialization of foods carrying “low-fat” claims, legally defined in the European Union by a fat threshold alone and with no accompanying requirement regarding the nutrients that replace it [2]. Such products are generally perceived by consumers as healthier alternatives and are frequently incorporated into dietary strategies aimed at weight management and chronic disease prevention [3]. Reducing fat from a processed food, however, requires substantial reformulation to preserve texture, flavor and palatability, and it is commonly replaced by refined starches, maltodextrins, rapidly digestible carbohydrates and free sugars. The resulting products contain less fat but not necessarily a healthier nutritional profile. Indeed, previous studies have shown that commercially available low-fat products frequently contain higher amounts of sugars and lower amounts of dietary fiber than their full-fat counterparts, despite presenting lower energy density [4,5]. The metabolic consequences of low-fat products may therefore depend less on the fat that has been removed than on the nutritional quality of the carbohydrates used to replace it. This concept has contributed to a growing recognition that evaluating diets exclusively according to macronutrient distribution may overlook important determinants of metabolic health.

Carbohydrates are the most heterogeneous macronutrient class, encompassing compounds that differ substantially in their chemical structure, digestibility and physiological fate. Digestible carbohydrates such as glucose, sucrose and maltodextrin contribute directly to postprandial glycemia and insulin secretion, whereas dietary fiber escapes digestion in the small intestine and is either fermented by the gut microbiota or excreted, exerting multiple beneficial effects on intestinal physiology, microbial metabolism and systemic metabolism [6]. Consequently, diets containing similar proportions of carbohydrates may induce markedly different metabolic responses depending on the relative contribution of dietary fiber, refined starches and free sugars. To address this limitation, the concept of carbohydrate quality has emerged as an important complementary indicator of dietary quality. Rather than considering only the total amount of carbohydrates consumed, carbohydrate quality reflects the nutritional characteristics of carbohydrate-containing foods, particularly their dietary fiber and free sugar content. Among the available metrics, the carbohydrate-to-fiber ratio (10:1) has become the most widely adopted indicator, recommending the presence of at least 1 g of dietary fiber for every 10 g of total carbohydrates [6,7]. More recently, modified indices incorporating free sugars have been proposed, including the 10:1:1 ratio, which additionally limits free sugars to less than 1 g per 10 g of carbohydrates [7]. These simple metrics can be applied at both the dietary-pattern and the individual food-product level and have shown consistent associations with healthier dietary patterns and with lower all-cause mortality [8].

Epidemiological evidence indicates that carbohydrate quality carries substantive implications for metabolic health. Higher adherence to favorable carbohydrate quality ratios has been associated with lower adiposity, reduced prevalence of obesity, improved insulin sensitivity and healthier body composition [9,10,11]. Likewise, better carbohydrate quality has been associated with a lower risk of metabolic dysfunction-associated steatotic liver disease (MASLD) [12,13,14], as well as chronic kidney disease (CKD) [14,15]. Notably, patients with MASLD have also been found to have nearly 40% higher long-term risk of developing CKD [16]. This clinical overlap is increasingly interpreted within the framework of the gut–liver–kidney axis, which provides a mechanistic rationale for the co-morbidity and synergistic progression of multi-organ metabolic disease [17]. The three organs are functionally coupled through a nutritional–metabolic continuum in which the availability of fermentable substrates shapes microbial community structure and, consequently, microbial metabolite output [18,19].

It is now well established that gut-derived microbial metabolites, including short-chain fatty acids (SCFAs) and precursors of uremic toxins such as indole, reach the liver via the portal circulation and subsequently enter the systemic circulation, where they modulate signaling pathways involved in metabolic homeostasis [20,21]. In the liver and kidney, for example, SCFAs regulate lipid metabolism, autophagic flux, and inflammatory responses [22,23,24]. Indole, generated through the bacterial fermentation of tryptophan, is absorbed in the colon and subsequently sulfated in the liver to form indoxyl-3-sulfate (I3S), a protein-bound uremic toxin that is cleared predominantly through tubular secretion [25]. Renal accumulation of I3S promotes glomerular and tubulointerstitial injury [26,27] and further exacerbates gut microbiota dysbiosis, thereby contributing to a self-perpetuating pathological loop [27]. These reciprocal interactions underscore the functional integration of the gut, liver, and kidney as a single interconnected axis, rather than as sequentially affected organs [28,29].

Because diet dictates the pool of substrates available for microbial fermentation, the contribution of hypercaloric dietary patterns rich in fat and sugar to dysbiosis and to deranged inter-organ crosstalk has been extensively documented [30,31]. Yet, whether isocaloric low-fat diets—typically fiber-depleted, free sugar-enriched and therefore of poor carbohydrate quality [32]—perturb the gut–liver–kidney axis remains unknown. Accordingly, the present work provides an integrated assessment of gut microbiota composition and fermentative output, together with structural, lipid-handling and injury-related responses in the liver and kidney, following chronic exposure to a low-fat diet. Specifically, it is investigated whether carbohydrate quality, independent of caloric load, is associated with alterations across the gut–liver–kidney axis. To this end, Wistar rats were exposed for 24 weeks to an isocaloric low-fat diet (LF), benchmarked against a high-fat diet (HF, positive comparator for diet-induced metabolic dysfunction) and an isocaloric whole-grain chow (reference).

## 2. Materials and Methods

### 2.1. Diets

**Figure 1:**
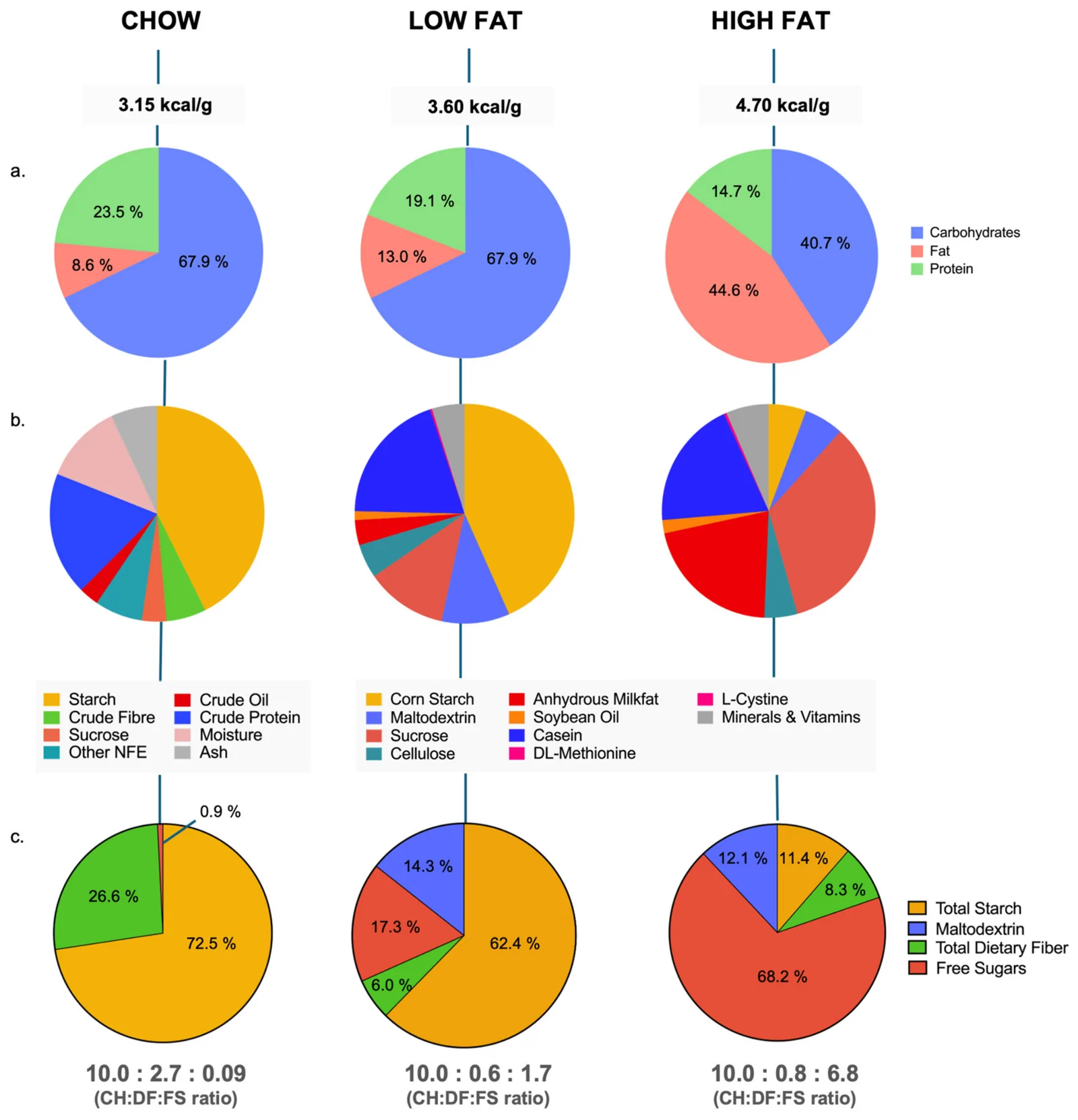
**Nutritional and carbohydrate quality characterization.** (**a**) Macronutrient composition to total energy (%kcal); (**b**) Gravimetric nutritional composition (g/100 g); (**c**) Carbohydrate-to-Dietary Fiber-to-Free Sugars (CH:DF:FS) ratio, with the corresponding proportions of total starch, total dietary fiber and free sugars within the carbohydrate fraction (% of total carbohydrate); Energy density (kcal/g) is indicated above each diet. Values in (**a**,**b**) are those declared by the manufacturers for each formulation and are therefore single values without replication. In (**c**), total dietary fiber and free sugars are the mean of three independent determinations per diet, whereas total starch is taken from the manufacturer’s specification, and maltodextrin is the balance of the carbohydrate fraction. CHOW, non-purified whole-grain reference diet of similar energy density; LF, purified isocaloric low-fat diet; HF, purified high-fat, high-sucrose diet.

Three commercially available diets were used. Two ingredient-matched purified diets were used as the isocaloric low-fat diet (LF; TD.08485, Inotiv, West Lafayette, IN, USA) and as the hypercaloric high-fat diet (HF; TD.08811, Inotiv, West Lafayette, IN, USA), and a whole-grain, non-purified chow diet (Chow; 4RF21, Mucedola, Settimo Milanese, Italy) was used as the isocaloric whole-grain reference. The nutritional composition and carbohydrate quality of the experimental diets are summarized in Figure 1. The energy density of each diet (kcal/g) is that declared by Inotiv and Mucedola. The modified carbohydrate-to-dietary fiber-to-free sugars ratio (CH:DF:FS ratio) was calculated according to the following formula:

10 g CH : [(DF × 10)/Total CH] : [(FS × 10)/Total CH],(1)

where total CH represents the sum (g) of dietary fiber, starch, sucrose, and maltodextrin, and FS represents the amount (g) of sucrose. Ratio terms are displayed rounded to the precision of the underlying determinations, and fold differences were calculated from the unrounded values. Starch content was taken from the manufacturers’ specifications and was not fractionated analytically. Maltodextrin was included in total carbohydrate but not in the free sugars term, as it is a digestible glucose polymer with a degree of polymerization ≥3 and therefore falls outside the definition of sugars (all monosaccharides and disaccharides present in food) of WHO free sugars, notwithstanding its rapid hydrolysis to glucose in vivo [33].

Ingredient-matched formulations differ by design in their fat and carbohydrate content, while chow differs from both purified diets in several additional dimensions: ingredient origin, protein source and content (plant protein versus casein supplemented with L-cystine and DL-methionine), fat source and degree of saturation, fiber type (mixed fermentable fibers and resistant starch versus poorly fermentable cellulose), phytate, phytochemical and micronutrient content, and degree of processing. The designations “low-fat” and “high-fat” refer to the fat content of the two purified formulations relative to each other, following the convention of the supplier’s matched diet pair, and not to their fat content relative to Chow. The HF diet is a high-fat, high-sucrose formulation and is referred to as such where that distinction matters.

#### 2.1.1. Determination of Dietary Fiber Content

Total dietary fiber content (g/100 g) was measured using the Megazyme Test Kit (Ref: K-TDFR; Megazyme International, Bray, Ireland), based on the Association of Official Analytical Chemists (AOAC) method 991.43, as previously described [34].

#### 2.1.2. Determination of Free Sugar Content

The free sugar composition was obtained by extraction following the methodology described by Ribeiro et al. (2020) [35]. Briefly, a sample (1 g) was extracted three times in succession with 25 mL of 80% (*v*/*v*) ethanol solution for 15 min at room temperature in an ultrasonic unit. Then, samples were centrifuged at 10,000× *g* for 10 min at 20° C. The supernatant was recovered, followed by solvent evaporation using a speed vacuum and subsequent dissolution in water. The HPLC analysis of the free sugar profile was performed using a Beckman Coulter System Gold HPLC (Knauer, Berlin, Germany) coupled to RI and using an Aminex HPX-87P column (Bio-rad, Hercules, CA, USA) at 85 °C and ultra-pure water as mobile phase at a flow rate of 0.6 mL min^−1^. The quantification of free sugars was achieved using standard calibration curves (0.1–40 mg/mL).

### 2.2. Animals and Experimental Design

An in vivo study was employed to provide an integrative assessment of inter-organ effects of low-fat diet across the gut–liver–kidney axis. Replacement was not feasible, as no in silico or in vitro system can reproduce multi-organ crosstalk over a 24-week exposure to dietary regimens. Twenty-four male healthy Wistar Han rats (13 weeks old, 380.4 ± 5.4 g) were housed in the animal facility of Coimbra Institute for Clinical and Biomedical Research (iCBR), Faculty of Medicine, University of Coimbra, two per cage (GR900, Tecniplast^®^ (Tecniplast S.p.A., Buguggiate, Italy)) on corncob bedding, with enriched Bio-Serv™ Rat Tunnels™ (Bio-Serv, Flemington, NJ, USA), under controlled environmental conditions with day/night cycles of 12 h, temperature 22 ± 1 °C and relative humidity 50–60% and with food and tap water supplied ad libitum (except during fasting periods). Cages were distributed across the holding rack with the three dietary groups represented at every shelf level, and cage position was rotated weekly to prevent systematic effects of cage location. Within each measurement session, animals were processed in a randomized order alternating between dietary groups, and all determinations for a given endpoint were performed in a single session by the same operator. Following an acclimation period of 2 weeks, the 12 cages were randomly allocated to the three experimental groups using Microsoft Excel. Random numbers were generated using the RAND() function, and the resulting randomized cage list was organized into three groups of four cages using the WRAPROWS function. The allocation was performed while maintaining comparable baseline body weights across the three groups. The groups were assigned to one of the following dietary regimens for 24 weeks: standard chow diet (Chow; n = 8), refined low-fat diet (LF; n = 8) or refined high-fat diet (HF; n = 8). Rats are a social species, and Directive 2010/63/EU recommends that such animals be housed in stable social groups; pair housing was therefore adopted as a welfare refinement, and the dietary treatment was necessarily allocated at the cage level. Accordingly, the cage was the experimental unit for dietary treatment allocation, whereas individual animals remained the observational units for animal-level measurements. The study was designed as an exploratory, broad phenotypic characterization, without a single confirmatory primary hypothesis. Reduction was addressed within sample-size planning, with body-weight gain over the 24-week intervention used as a reference outcome. An a priori sample size calculation was performed using G*Power for macOS, Version 3.1.9.6 for a one-way ANOVA (fixed effects, omnibus), assuming an effect size of f = 0.80, derived from the between-group difference in body-weight gain previously obtained in our laboratory under the same high-fat feeding protocol [34], an α error probability of 0.05, a statistical power of 0.90, and three experimental groups. The analysis indicated a required total sample size of 24 animals (n = 8 per group), corresponding to an actual power of 0.913. The calculation was based on the assumption of independent animal-level observations and did not incorporate an adjustment for potential within-cage correlation. Group allocation was performed by an independent researcher. Personnel responsible for administering dietary treatments and conducting experimental procedures were aware of group allocation. However, outcome assessment and data analysis were performed by researchers blinded to group allocation. The allocation code was only broken after completion of data analysis.

All 24 animals completed the 24-week intervention, and no animal was excluded from the study. Exclusion criteria were defined at two distinct levels. At the level of the animal, the humane endpoints described above were the only predefined criteria for removal from the study, and no further animal-level exclusion criterion was applied. At the level of the individual measurement, outlying values were identified by the predefined ROUT procedure described in Section 2.13. The number of animals contributing to each analysis is compiled, endpoint by endpoint, in Supplementary Table S1, which also gives the reason for every departure from the initial n = 8 per group. Such departures arose only from (i) exclusion of individual values identified as statistical outliers, (ii) endpoints performed in a predefined subset of animals because of the throughput of the technique and/or (iii) failure/unavailability of an individual sample. Individual data points identified as statistical outliers were excluded from the corresponding endpoint-specific analysis, while the respective animals remained included in all other analyses for which valid data were available.

Animal health and welfare were monitored daily by trained animal facility personnel throughout the experimental period, with particular attention to general appearance, behavior, locomotor activity, food and water intake, and signs of pain or distress. In addition, body weight (BW) and food and water consumption were recorded weekly by the research team. Because animals were pair-housed, food and water consumption were measured per cage and divided by the number of animals in the cage; intake data therefore represent the cage mean. Humane endpoints included a body weight loss greater than 20% and persistent inability to eat or drink; animals reaching any of these criteria would have been removed from the study and humanely euthanized. No animal reached the predefined humane endpoints, and no mortality or unexpected adverse events occurred during the study. No routine analgesia was administered during the experimental period, as the procedures performed in conscious animals did not involve surgery and were expected to cause only transient mild discomfort. Terminal procedures were performed under deep anesthesia after fasting periods.

At the end of the study, animals were anesthetized in a saturated chamber with isoflurane (IsoFlo^®^, Abbott, Abbott Park, IL, USA) followed by intraperitoneal injection of ketamine chloride (150 mg/kg; 100 mg/mL; Imalgene^®^, Boehringer Ingelheim, Lyon, France) in chlorpromazine (5 mg/kg; 25 mg/mL; Largactil^®^, Faes Farma Portugal, S.A., Amadora, Portugal). Adequate anesthetic depth was confirmed before cardiac puncture by the absence of pedal withdrawal and corneal reflexes. Death was subsequently confirmed by the absence of spontaneous respiration, heartbeat, and reflex responses following blood collection and perfusion. Blood was immediately collected through heart puncture into serum tubes (BD Vacutainer SST II 47 Advance, Fisher Scientific, Porto Salvo, Portugal) and then centrifuged at 3500 rpm for 15 min (4 °C) and stored at −20 °C. Upon sacrifice, rats were transcardially perfused with ice-cold PBS1x and kidneys, liver, and epididymal adipose tissue (EAT) were isolated, washed, and properly stored for further analysis. The experiment was carried out in strict compliance with the National and European Communities Council Directives of Animal Care and with the ARRIVE guidelines for reporting animal research [36].

### 2.3. Metabolic Characterization

#### 2.3.1. Glucose Tolerance Test and Insulin Tolerance Test

At the beginning and end of week 24, all animals were submitted to a GTT and ITT, respectively. After a 6 h fasting period, rats were injected intraperitoneally with a glucose solution (Sigma-Aldrich, St. Louis, MO, USA, 2.0 g/kg BW) for GTT or with a saline injection of insulin (Novo Rapid, Novo Nordisk^®^, Bagsværd, Denmark, 0.75 U/kg BW) for ITT. Blood samples were collected from the tail vein: the first drop of blood was discarded, and the second drop was used to measure baseline glucose levels. Serum glucose measurements occurred at subsequent time points following glucose or insulin administration (GTT assay: 15, 30, 60, 120 min; ITT assay: 15, 30, 45, 60, 120 min) at selected time points. The glucose levels were measured using a portable commercial glucometer kit (GlucoMen^®^ aero 2K, A. MENARINI diagnostics, Florence, Italy; declared measuring range 20–600 mg/dL). The AUC of GTT and of ITT curves were calculated using the trapezoidal method, as previously described [37]. Tail-vein blood was obtained by needle puncture without local anesthesia, as the procedure is brief and the additional handling required for topical anesthesia would itself have been a greater source of distress; a single drop (approximately 5 µL) was collected at each time point, corresponding to less than 100 µL per animal per test and to well below 1% of the circulating blood volume.

Throughout the ITT, animals were closely monitored for clinical signs of hypoglycemia, including tremor, reduced activity, abnormal posture, lethargy, and impaired responsiveness. Based on previously described procedures [38,39], rescue criteria were defined as a blood glucose concentration below 20 mg/dL or the occurrence of clinical signs compatible with hypoglycemia, irrespective of the measured blood glucose concentration. If either criterion was met, the ITT was immediately discontinued, and a 10% glucose solution (200 mg/kg; 2 mL/kg, i.p.; Sigma-Aldrich, Merck) was administered, followed by free access to food and continued monitoring until recovery. Data collected after rescue intervention were excluded from the ITT analysis. No animal required glucose rescue during the present study.

Metabolizable energy, fecal energy loss, energy expenditure, physical activity and body composition were not assessed.

#### 2.3.2. Glucose and Insulin Levels

Blood samples used to determine non-fasted glucose/insulin levels were collected from the sacrifice day, under anesthesia, whereas fasting glucose and insulin levels were determined in conscious animals on the day of the ITT (week 24) at time 0 using capillary tubes (Microvette^®^ CB 300Z, Ref. 16.440). Collected blood was immediately centrifuged (1500× *g*, 10 min, 4 °C) for serum and insulin determinations. Serum glucose levels were measured with the aid of the portable commercial glucometer kit (GlucoMen^®^ aero 2K, A. MENARINI diagnostics, Florence, Italy), while serum insulin levels were quantified by a rat insulin ELISA (Enzyme-Linked Immuno-Sorbent Assay) kit from Mercodia (Uppsala, Sweden), according to the manufacturer’s kit procedure. From the fasting values, insulin resistance and insulin sensitivity were estimated as HOMA-IR = [fasting insulin (μU/mL) × fasting glucose (mmol/L)]/22,5 and QUICKI = 1/[log fasting insulin (μU/mL) + log fasting glucose (mmol/L)], with fasting insulin converted from μg/L to pmol/L by multiplying by 172.18 and subsequently to μIU/mL by dividing by 6.945, and fasting glucose converted from mg/dL to mmol/L by multiplying by 0.0555; both indices have been validated against the hyperinsulinemic–euglycemic clamp in Wistar rats [40].

#### 2.3.3. Triglyceride Profile Evaluation

Serum levels of triglycerides (TGs), low-density lipoprotein cholesterol (LDL-c), high-density lipoprotein cholesterol (HDL-c), and total cholesterol were evaluated by routine procedures through automatically validated methods using a Chemistry Analyzer (Hitachi 717 analyzer, Roche Diagnostics GMBH, Mannheim, Germany), as previously described [40].

Triglyceride contents in kidney and liver samples were measured by an enzymatic colorimetric assay using a commercial kit (Ref.1155010, Triglycerides MR, Cromatest^®^, Linear Chemicals, Barcelona, Spain). Briefly, 50 mg of frozen tissue was homogenized in 1 mL of isopropanol. The homogenate was sonicated and then centrifuged at 3000 rpm for 5 min at 4.°C, and the supernatant was analyzed following the manufacturer’s instructions.

### 2.4. Serum and 24 h Urine Parameters

Albumin and creatinine in serum were evaluated by validated automated methods and equipment (Hitachi 717, Roche Diagnostics Inc., MA, USA). At week 22, animals were individually housed in metabolic cages for a 24 h urine and feces collection period. At the end, serum glycemia was measured by the aid of the portable commercial glucometer kit (GlucoMen^®^ areo 2K, A. MENARINI diagnostics, Florence, Italy). No separate acclimatization period to the metabolic cages was performed before sample collection. During the collection period, animals were maintained under the same controlled environmental conditions used in the home cages (12 h light/dark cycle, 22 ± 1 °C, and 50–60% relative humidity), with food and water provided ad libitum. No bedding was used in the metabolic cages to avoid contamination of urine and fecal samples. Creatinine was quantified by the colorimetric compensated Jaffe method and albumin by immunoturbidimetry, the same chemistry being applied to serum and urine samples.

Urinary glucose, creatinine and albumin concentrations were evaluated in 24 h urine samples (Cobas Integra 400 plus, Roche^®^, Amadora, Portugal), and urine volumes were measured to calculate glucose clearance and albumin/creatinine ratio. ACR was calculated by the following formula:

ACR (mg/g) = [Urinary Albumin (mg/dL)/Urinary Creatinine (mg/dL)] × 1000(2)

### 2.5. Epididymal Adipose Tissue, Liver and Kidney Histomorphology

#### 2.5.1. Hematoxylin–Eosin (H&E) Staining

EAT and liver samples were formalin-fixed and embedded in paraffin wax for hematoxylin–eosin (H&E) staining. Cryosections (5 µm) from each block were reviewed. Briefly, tissue sections were deparaffinized in xylene and hydrated to a decreasing series of ethanol until distilled water. Thereafter, the tissue sections were immersed in hematoxylin stain Solution, Gill 1 (Sigma Aldrich, Saint Louis, MO, USA) for 2 min and washed in tap water. Then, they were counterstained with 0.5% aqueous eosin (Sigma Aldrich, St. Louis, MO, USA) for 30 s and after that dehydrated, cleared, and mounted. All samples were examined by light microscopy using a Zeiss microscope Mod. Axioplan 2 (Göttingen, Germany), as previously described [34].

#### 2.5.2. Periodic Acid of Shiff (PAS) Staining

For PAS staining, kidney sections were deparaffinized in xylene and hydrated to a decreasing series of ethanol until distilled water. Then, they were treated with periodic acid (Sigma Aldrich, Saint Louis, MO, USA) for 5 min and rinsed in distilled water. After that, they were stained with Schiff’s reagent (Sigma Aldrich, Saint Louis, MO, USA) for 10 min and washed in tap water for 3 min. Nuclei were counterstained with hematoxylin stain Solution, Gill 1 (Sigma Aldrich, Saint Louis, MO, USA) for 1 min and rinsed in tap water. Tissue sections were then dehydrated, cleared and mounted. All samples were examined by light microscopy using a Zeiss microscope Mod. Axioplan 2 (Göttingen, Germany), as previously reported [41].

#### 2.5.3. Gomori’s Green Trichrome Staining

Kidney sections were deparaffinized in xylene and hydrated to a decreasing series of ethanol until distilled water. Then the slides were treated with Weigert’s iron hematoxylin (Sigma Aldrich, Saint Louis, MO, USA) stain for 7 min. Slides were placed into Chromotrope 2R (Sigma Aldrich, Saint Louis, MO, USA) at 0.2% with Light green at 0.8% solution for 5 min. Then, a quick rinse of the stain with distilled water was performed, and then it was differentiated with acetic acid at 0.5%. Tissue sections were then dehydrated, cleared, and mounted. The cytoplasm of the cells was stained in red and fibrin in pink, whereas collagen was observed in green, in contrast with blue or black nuclei. All samples were examined by light microscopy using a Zeiss microscope Mod. Axioplan 2 (Göttingen, Germany), as previously reported [41].

### 2.6. Immunohistochemistry

Cross-sections (10 µm thickness) of rat kidney were cut with a cryostat (Leica CM3050S, Nussloch, Germany). Kidney cryosections were fixed with an acetone:methanol mixture (85%/15%) at 20 °C for 10 min and then rehydrated in PBS (3 × 5 min). After washing, sections were permeabilized with 0.35% Triton X-100 in PBS for 15 min and blocked for 40 min with 10% fetal bovine serum (FBS) in PBS. The sections were incubated with primary antibody (1:100) mouse anti-synaptopodin (sc515842; Santa Cruz Biotechnology, Dallas, TX, USA) in PBS containing 3% FBS overnight at 4 °C. After rinsing with PBS (3 × 5 min), the sections were incubated with the secondary fluorescent antibody Alexa Fluor 568-conjugated donkey anti-mouse IgG (1:500; Molecular Probes, Life Technologies, Paisley, UK) and 4’,6-diamidino-2-phenylindole (1:5000; DAPI, Sigma-Aldrich, Merck, Darmstadt, Germany) for 1 h at room temperature. After incubation, the sections were washed with PBS (3 × 5 min), and the slides were mounted using the Glycergel mounting medium (Dako, Carpinteria, CA, USA). Immunostained samples were imaged using a confocal fluorescence microscope (LSM 710, Carl Zeiss, Gottingen, Germany).

### 2.7. BODIPY^®^ Fluorescence Staining

For the BODIPY staining, cross-sections (10 µm thickness) of liver tissue were washed (3 × 5 min) in phosphate-buffered saline (PBS) and incubated with 0.5 µg/mL of BODIPY^®^ 493/503 (#790389, Sigma-Aldrich, Merck, Darmstadt, Germany) in PBS for 30 min at room temperature. After washing, the cell nuclei were stained with 4′,6-diamidino-2-phenylindole (1:5000; DAPI, Sigma-Aldrich, Merck, Darmstadt, Germany) for 15 min at room temperature. After incubation, the sections were washed with PBS (3 × 5 min), and the slides were mounted using Glycergel mounting medium (Dako, Carpinteria, CA, USA). Images were captured using a confocal fluorescence microscope (LSM 710, Carl Zeiss, Gottingen, Germany), as previously reported [42].

### 2.8. Image Analysis and Data Quantification

#### 2.8.1. Epididymal White Adipose Tissue

Five to ten high-resolution images per animal, with 6 to 8 animals per group, were captured by an Axiocam 105 color (Zeiss, Jenna, Germany) camera under the same parameter settings for adipocyte morphometry analysis, using a Zeiss microscope Mod. Axioplan 2 (Zeiss, Jenna, Germany). Slides were imaged at 10x magnification. To quantify the mean adipocyte area and distribution, an average of 512 adipocytes per animal were analyzed using the FIJI (ImageJ v2) plugin Adiposoft. Images were captured and imported into ImageJ software and analyzed by the ImageJ plugin Adiposoft. To ensure that only adipocytes were counted, each image was manually inspected. The artifacts and broken adipocytes that were unduly analyzed and accounted for by the software were recorded and eliminated manually from the output Excel sheet that contained the diameter and area data of all the adipocytes in each image. The mean adipocyte area and mean adipocyte diameter were calculated for each animal and for each group.

#### 2.8.2. Liver

Two high-resolution images per animal, with 6 to 8 animals per group, imaged at 40x magnification were captured under the same parameter settings for hepatocyte morphometry analysis using a Zeiss microscope Mod. Axioplan 2 (Zeiss, Jena, Germany). To evaluate the liver morphology and the degree of steatosis, approximately 200 hepatocytes per animal were analyzed using the FIJI (ImageJ v2) plugin Cell Counter. Each acquired image was imported to ImageJ and analyzed individually. The number of total hepatocytes in each image was calculated by manually counting all the visible hepatocyte nuclei.

To assess the total steatosis score (%), the ratio of the total number of steatotic hepatocytes and the total number of hepatocytes times 100 was calculated according to the following formula:

Total Steatosis Score (%) = (Microsteatotic + Macrosteatotic hepatocytes)/Total number of hepatocytes × 100(3)

#### 2.8.3. Kidney Tissue

Kidney sections stained for synaptopodin from 6 to 8 animals per group were examined using a confocal fluorescence microscope (LSM 710, Carl Zeiss, Gottingen, Germany) and analyzed using FIJI (ImageJ v2). Images were acquired at 20× magnification under identical acquisition settings. For each animal, eight non-overlapping fields were selected from spatially opposed regions of the kidney section in order to provide representative sampling across the entire renal tissue, with a minimum of 20 glomeruli analyzed per animal. Images acquired in ZeissVisionImage (.zvi) format were exported as TIFF files. The channels of the original RGB images were separated, and the red channel corresponding to synaptopodin staining was selected for analysis. A duplicate image of the Red channel was made and convoluted using a Gaussian Blur filter. Next, the brightness and contrast features of the corrected image were manually adjusted using the “B/C Adjustment” (Brightness and Contrast) tool to further enhance the foreground. Subsequently, the adjusted image was thresholded (with Huang algorithm), and a binary mask was created. In addition, the binary operation “Fill Holes” was applied to fill intra-aggregate spaces, and the regions of interest (ROIs) were created. The ROIs defined by the binary mask were redirected to the original image, and the parameter’s integrated density, corresponding to fluorescence intensity and two-dimensional (2D) area, was determined. Brightness and contrast adjustments were applied exclusively to generate the binary mask used to define the regions of interest; all measurements were performed on the original, unadjusted images, to which the regions of interest were redirected. Identical acquisition settings were applied to all sections, and the analysis was performed blinded to dietary group. Glomerular area was not measured, and the synaptopodin-immunostained area is therefore reported per glomerulus without normalization to glomerular area.

### 2.9. Transmission Electron Microscopy (TEM)

Immediately after collection, renal tissue was cut into fragments of approximately 1 mm^3^ and immersed in 2.5% glutaraldehyde prepared in 0.1 M phosphate buffer (pH 7.2) for 2 h. Post-fixation was then carried out in 1% osmium tetroxide for 1.5 h, followed by contrast enhancement with 1% aqueous uranyl acetate for 1 h in the dark. Following several rinses in distilled water, the fragments were dehydrated through an ascending acetone series (30–100%) and infiltrated with an epoxy resin (Fluka Analytical, Sigma-Aldrich, Darmstadt, Germany). Ultrathin sections were cut on a Leica EM UC6 ultramicrotome (Leica Microsystems, Vienna, Austria), collected onto copper grids and contrasted with 0.2% lead citrate for 10 min. Grids were examined in a Tecnai™ G2 Spirit BioTWIN transmission electron microscope (FEI, Hillsboro, OR, USA) operated at an accelerating voltage of 100 kV, and the acquired micrographs were processed with AnalySIS 3.2 software. Ultrastructural analysis was qualitative: 3 animals per group and 10 glomeruli per animal were examined, and no morphometric quantification of glomerular basement membrane thickness or of foot-process width and spacing was performed.

### 2.10. Assessment of Gut Microbiota Composition and Function

#### 2.10.1. Real-Time PCR for Microbial Compositional Analysis of Stool

Genomic DNA was extracted and purified from fecal samples using NZY Tissue gDNA Isolation Kit (Nzytech^®^, Ref. MB135, Lisboa, Portugal) with some modifications [37]. Briefly, the feces (170–200 mg) were homogenized in TE buffer (10 mM Tris/HCl; 1 mM EDTA, pH 8.0) and centrifuged at 4000× *g* for 15 min. The supernatant was discarded, and the pellet was resuspended in 1 mL of buffer NT1. Then, 25 μL of proteinase K was added to 200 μL of the supernatant for incubation at 56 °C for 1 h and 45 min. The remaining steps followed the manufacturer’s instructions. DNA purity and quantification were assessed with a NanoDrop spectrophotometer (Thermo Scientific, Wilmington, DE, USA). Real-time PCR was performed in sealed 96-well microplates using an iQ™ SYBR^®^ Green Supermix (Bio-rad, Hercules, CA, USA) and a Bio-Rad CFX96 Connect Real-time PCR System instrument (Bio-Rad Laboratories, Hercules, CA, USA). PCR reaction mixtures (total of 10 μL) contained 5 μL of iQ™ SYBR^®^ Green (Bio-rad, Hercules, CA, USA), 1 μL of each primer (final concentration of 0.1 μM), 2 μL of water, and 1 μL of DNA (equilibrated to 20 ng). Primer sequences (STAB VIDA, Lisboa, Portugal) used to target the 16S rRNA gene of the bacteria and the conditions for PCR amplification reactions are listed in Table 1. Data were processed and analyzed using the CFX Maestro software, version 2.0 (BioRad Laboratories). Standard curves were constructed using serial tenfold dilutions of bacterial genomic DNA, according to the following webpage https://www.thermofisher.com/jp/ja/home/brands/thermo-scientific/molecular-biology/molecular-biology-learning-center/molecular-biology-resource-library/thermo-scientific-web-tools/dna-copy-number-calculator.html, accessed on 19 May 2021. Bacterial genomic DNA used as a standard (Table 1) was obtained from DSMZ (Braunschweig, Germany). Genome size and the copy number of the 16S rRNA gene for each bacterial strain used as a standard were obtained from the NCBI Genome database (www.ncbi.nlm.nih.gov). Data are presented as the mean values of duplicate PCR analysis. The F:B ratio was obtained by dividing the number of copies of Firmicutes division by the number of copies of Bacteroidetes division.

#### 2.10.2. Assessment of Indoxyl-3-Sulfate

Urinary indoxyl-3-sulfate (I3S, Indican) concentrations were quantified with the Indican Assay Kit (MAK128, Sigma-Aldrich, St. Louis, MO, USA), following the protocol reported previously [49]. In summary, each urine sample (50 μL) was placed into a well of a clear 96-well plate and combined with 140 μL of Reagent A. After gentle mixing, a baseline absorbance reading was taken at 480 nm (A480). Reagent B (10 μL) was then added to each well; the plate was mixed again and left to incubate for 5 min at room temperature, after which absorbance was recorded a second time to obtain the sample value. For the standard curve, wells containing 10 μL of the kit-supplied standard (30 mg/dL) were prepared and analyzed under the same conditions. Indican concentration was calculated using the formula provided by the manufacturer:

(4) $$\mathrm{Indican}\left(\mathrm{mg}/\mathrm{dL}\right)=\frac{\left(A480_{sample}-A480_{blank}\right)\;\times 5\;\times \;n}{A480_{standard}-A480_{sample}}$$

where *n* represents the sample dilution factor. If calculated values exceeded 20 mg/dL, samples were diluted in water and reanalyzed, with the final concentration adjusted for the dilution factor. All assays were performed in duplicate, and absorbance was read using a spectrophotometric microplate reader set at 480 nm.

#### 2.10.3. Fecal SCFAs Determination

SCFA concentrations were measured using the method described by Scortichini et al. [50]. Thawed stool samples were homogenized; then, 100 mg of feces were weighed. The stool sample was acidified with 0.25 mL of sulfuric acid (50%) and shaken for 3 min. Then, 1 mL of diethyl ether was added to the acidic solution to extract the SCFAs. The sample was centrifuged for 5 min at 2800× *g*, and the organic phase was collected in another vial. The extraction was repeated three times. Finally, 0.5 µL of the harvested organic phase was injected into a gas chromatograph (GC) for analysis. The individual acids were quantified by gas chromatography equipped with a flame ionization detector (Agilent 7890 series II Agilent Technologies, Santa Clara, CA, USA) using a BPX 70 column (BPX70, 25 m × 0.22 mm ID × 0.25 μm, SGE Analytical Science, Ringwood, Australia). Quantification was achieved by using calibration curves, and peak integration was performed using MSD ChemStation (Agilent Technologies, Santa Clara, CA, USA). Acid concentrations are expressed in [μmol/g].

### 2.11. Gene Expression by Quantitative Real-Time PCR Analysis

RNA was extracted from 30 mg of samples using the NZY Total RNA Isolation Kit (#MB13402; Nzytech, Lisboa, Portugal). RNA concentration and purity (A260/A280) were determined by spectrophotometry on the NanoDrop^®^ ND-1000 Spectrophotometer (Thermo Fisher Scientific, Wilmington, DE, USA). Synthesis of complementary Deoxyribonucleic acid (cDNA) was performed using the iScript Select cDNA Synthesis Kit (#1708891; Bio-Rad, Hercules, CA, USA) following the manufacturer’s instructions. For each PCR reaction, a 20 μL volume was used containing 2 μL of cDNA, 10 μL of Sybr Green (iTaq Universal SYBR Green Supermix 1725124, Bio-Rad, Hercules, CA, USA), 0.4 μL of each primer (listed in Table 2) and 7.6 μL of autoclaved DEPC water. Gene expression was normalized using the geNorm algorithm [n], which ranked β-actin and hypoxanthine-guanine phosphoribosyltransferase (HPRT) as the most stable pair among the candidate reference genes evaluated, with an expression stability value M = 0.955, below the M < 1.5 threshold proposed for acceptable reference gene stability. The relative expression ratio of each target gene was computed based on ΔΔCt (2^−ΔΔCt^). Results are expressed as percentage of control.

### 2.12. Protein Expression by Western Blotting

#### 2.12.1. Protein Extraction and Quantification

Approximately 50 mg of liver and kidney was homogenized by mechanical dissociation using a handheld tissue homogenizer in a cold (4 °C) radioimmunoprecipitation assay (RIPA) lysis buffer containing 50 mM Tris-HCl (pH 8.0), 150 mM NaCl, 0.1% (*v*/*v*) sodium dodecyl sulphate (SDS), 1% (*v*/*v*) Triton X-100, and 5 mM ethylenediaminetetraacetic acid (EDTA), supplemented with 1 pill of protease inhibitor cocktail (11836170001, Roche Diagnostics GmbH, Germany), 1 mM of phenylmethylsulfonyl fluoride (PMSF) and 1 mM of sodium orthovanadate (Na_3_VO_4_), as previously described [51]. Homogenates were left on ice for 1 h and vortexed every 15 min. Sample lysates were centrifuged for 20 min at 13,000 rpm at 4 °C. After centrifugation, the supernatant fractions were collected and centrifuged again. After the last centrifugation, the supernatant fractions were collected, and the protein lysates of each sample were used to determine the protein concentration. The remaining samples were stored at −80 °C. The protein concentration was determined using the bicinchoninic acid (BCA) assay (Pierce™ BCA Protein Assay Kit, Pierce Biotechnology, Rockfor, IL, USA) using bovine albumin serum (BSA) standard solutions. Absorbance readings were performed at 570 nm using BioTek Synergy™ HT and Gen5 Data Analysis Software after the plate was incubated for 30 min at 37 °C. The total protein concentration in each sample was calculated using the calibration curve obtained from the BCA assay.

#### 2.12.2. Polyacrylamide Gel Electrophoresis and Immunodetection

Denaturation was carried out by combining protein extracts with a 6× concentrated loading buffer (0.35 M Tris-HCl, pH 6.8; 30% (*v*/*v*) glycerol; 0.65 M dithiothreitol; 10% (*w*/*v*) SDS; 0.03% (*w*/*v*) bromophenol blue) and heating the mixture at 95 °C for 5 min, following the procedure reported previously [34]. Aliquots corresponding to 30 µg of hepatic or renal protein were applied to each lane and resolved on 10–15% polyacrylamide gels under denaturing conditions (SDS-PAGE). Electrophoresis was conducted at 4 °C in running buffer (125 mM Tris base, 950 mM glycine, 0.5% (*w*/*v*) SDS, pH 8.3), initially at 70 V for 20 min and subsequently at 100 V for a further 150–210 min; run duration was adjusted for each target according to its molecular weight, using the migration of a prestained standard (Protein Marker VI, 10–245 kDa; A8889.0500, AppliChem GmbH, Darmstadt, Germany) as reference. Resolved proteins were subsequently transferred onto 0.45 µm PVDF membranes (Amersham™ Hybond™, GE Healthcare, Buckinghamshire, UK), previously activated, by electroblotting at 100 V for 90 min at 4 °C in a CAPS-based transfer buffer (100 mM *N*-cyclohexyl-3-aminopropanesulfonic acid, pH 11). Transfer efficiency and homogeneity of protein loading across lanes were verified by reversible Ponceau S staining; the dye was subsequently removed with 0.1 M NaOH, followed by washing in Tris-buffered saline supplemented with 0.1% (*v*/*v*) Tween-20 (TBS-T; 150 mM NaCl, 20 mM Tris-HCl, pH 7.6). Membranes were then saturated for 1 h at room temperature under gentle agitation, using either non-fat dry milk or bovine serum albumin diluted in TBS-T, as specified in Table 3. Incubation with the corresponding primary antibodies (Table 3) proceeded overnight at 4 °C with agitation. Membranes were rinsed in TBS-T, exposed to the appropriate secondary antibodies (Table 3) for 1 h at room temperature with agitation, and rinsed again in TBS-T. Immunoreactive bands were revealed with enhanced chemiluminescence reagents—WesternBright™ ECL (R-03031-D25) or WesternBright™ Sirius (R-03027-C50), each combined with the respective peroxide solution (R-03025-D25 and R-03025-C50; Advansta, San Jose, CA, USA)—and acquired on an ImageQuant™ LAS500 imaging system (GE Healthcare, Buckinghamshire, UK). Loading was further controlled by reprobing the same membranes for the housekeeping protein glyceraldehyde 3-phosphate dehydrogenase (GAPDH, Table 3). Band intensities were determined by densitometric analysis in ImageJ (LOCI, University of Wisconsin, Madison, WI, USA), normalized to the respective loading control, and reported as a percentage of the control group.

### 2.13. Statistical Analysis

The distribution of continuous variables was analyzed using the Shapiro–Wilk test to assess significant deviations from normality. Given the group sizes, this test was used to screen for departures from normality rather than to establish it, and the nonparametric route was adopted whenever a departure was detected. Differences between experimental groups were compared using the nonparametric Kruskal–Wallis test (followed by Dunn’s test for multiple comparisons) for non-normally distributed data or the parametric test one-way ANOVA, followed by Bonferroni’s test for multiple comparisons for normally distributed data. Repeated measures ANOVA, followed by the Bonferroni post hoc test, was used to compare BW evolution during the experimental period and glucose levels throughout the GTT and ITT assays. Statistical outliers were identified using the ROUT (robust regression and outlier removal) method in GraphPad Prism, with the false discovery rate set at Q = 1%. Outlier assessment was performed separately for each outcome, and identified values were excluded only from the corresponding analysis. This procedure operates at the level of individual measurements and was not used as a criterion for excluding animals. 

Results are depicted as the mean standard error of the mean (SEM), and data analyses were performed using GraphPad Prism software for macOS, version 9.5.0 (730) (GraphPad Software, Inc., La Jolla, CA, USA). A *p* value < 0.05 was considered statistically significant. No correction was applied for the number of endpoints examined. Body-weight gain was the outcome on which the a priori sample-size calculation was based; beyond it, no endpoint was designated as primary, and the endpoints were selected to characterize a phenotype rather than to test a confirmatory hypothesis. Both refined diets were compared with the Chow reference and with each other.

## 3. Results

### 3.1. Nutritional and Carbohydrate Quality Characterization

The three experimental diets were characterized in terms of macronutrient distribution, ingredient origin and carbohydrate quality (Figure 1). In terms of energy density, the Chow and LF diets provided 3.15 and 3.60 kcal/g, respectively, values consistent with their classification as isocaloric regimens, whereas the HF diet, energy-dense by formulation, provided 4.70 kcal/g. Regarding the contribution of each macronutrient to total energy (Figure 1a), Chow provided 67.9% of energy as carbohydrate, 23.5% as protein and 8.6% as lipid. The LF diet delivered a carbohydrate contribution superimposable on that of (67.9%), with a lower energy contribution from protein (19.1%) offset by a higher lipid contribution (13.0%); the HF diet, by contrast, derived 44.6% of its energy from lipid, 40.7% from carbohydrate and 14.7% from protein. In this respect, it should be emphasized that the “low-fat” designation corresponds to the operational definition applied to processed foods and holds relative to its ingredient-matched high-fat counterpart; it does not, however, denote a lower fat content than the whole-grain control. Qualitatively, the Chow diet was manufactured exclusively from whole-grain ingredients, whereas both processed regimens, LF and HF, were formulated from refined ingredients. The corresponding gravimetric composition of each diet is depicted in Figure 1b. Chow and the purified diets differ in kind rather than in degree. Chow is manufactured from whole-grain cereals, so that its protein is of plant origin, its carbohydrate is delivered as native starch within an intact grain matrix, and its fiber fraction comprises mixed fermentable fibers together with resistant starch; the same matrix also carries phytate, phytochemicals and grain-derived micronutrients. In the purified diets, each macronutrient is supplied by a single defined ingredient: protein as casein supplemented with L-cystine and DL-methionine, carbohydrate as corn starch, maltodextrin and sucrose, fiber exclusively as cellulose, lipid as anhydrous milkfat with soybean oil, and micronutrients as defined mineral and vitamin premixes. LF and HF share this ingredient list and differ only in the proportions in which sucrose and anhydrous milkfat replace corn starch.

Although Chow and LF supplied an identical proportion of energy as carbohydrate, the internal composition of this fraction differed substantially (Figure 1c). Expressed as a carbohydrate-to-dietary fiber-to-free sugar (CH:DF:FS) ratio, Chow presented a profile of 10.0:2.7:0.09, denoting a fiber-rich carbohydrate fraction virtually devoid of free sugars. By contrast, the LF diet presented a ratio of 10.0:0.6:1.7, corresponding to a 4.4-fold lower dietary fiber content and a 19-fold higher free sugar content per unit of carbohydrate, whereas the HF diet presented a ratio of 10.0:0.8:6.8, i.e., a 3.2-fold lower fiber content and a 76-fold higher free sugar content than Chow, and a 3.9-fold higher free sugar content than the LF diet. Neither refined diet meets the 10:1 carbohydrate-to-fiber threshold, and both exceed the free-sugar limit of the 10:1:1 criterion.

### 3.2. Effects of Low-Fat Diet on Body Weight and Adiposity

Food and beverage consumption and the resulting caloric intake are summarized in Table 4. Both processed diets were associated with a lower food intake than Chow (LF: 116.81 ± 3.31 g/week; HF: 104.79 ± 4.24 g/week vs. Chow: 146.09 ± 1.73 g/week; *p* < 0.001 for both), whereas beverage intake was significantly reduced only in HF animals (175.80 ± 4.82 vs. 200.11 ± 2.17 mL/week; *p* < 0.001). Despite this lower food intake, total caloric intake in the LF group was only modestly reduced relative to Chow (420.51 ± 11.93 vs. 460.18 ± 5.45 kcal/week; *p* < 0.01), whereas HF animals ingested significantly more calories than LF animals (492.49 ± 19.93 kcal/week; *p* < 0.01 vs. LF), consistent with the higher energy density of that formulation. The distribution of ingested calories mirrored diet formulation: LF animals obtained slightly fewer calories from carbohydrate (285.51 ± 8.10 kcal/week; *p* < 0.05 vs. Chow) and more from lipid (54.66 ± 1.55 kcal/week; *p* < 0.001 vs. Chow) than Chow animals, whereas HF animals showed the expected pronounced increase in lipid-derived calories (219.65 ± 8.89 kcal/week; *p* < 0.001 vs. Chow and vs. LF) at the expense of carbohydrate-derived calories (200.43 ± 8.12 kcal/week; *p* < 0.001 vs. Chow and vs. LF). Protein-derived calories were reduced in both processed groups (LF: 80.33 ± 2.28; HF: 72.40 ± 2.93 vs. Chow: 108.14 ± 1.28 kcal/week; *p* < 0.001).

**Figure 2:**
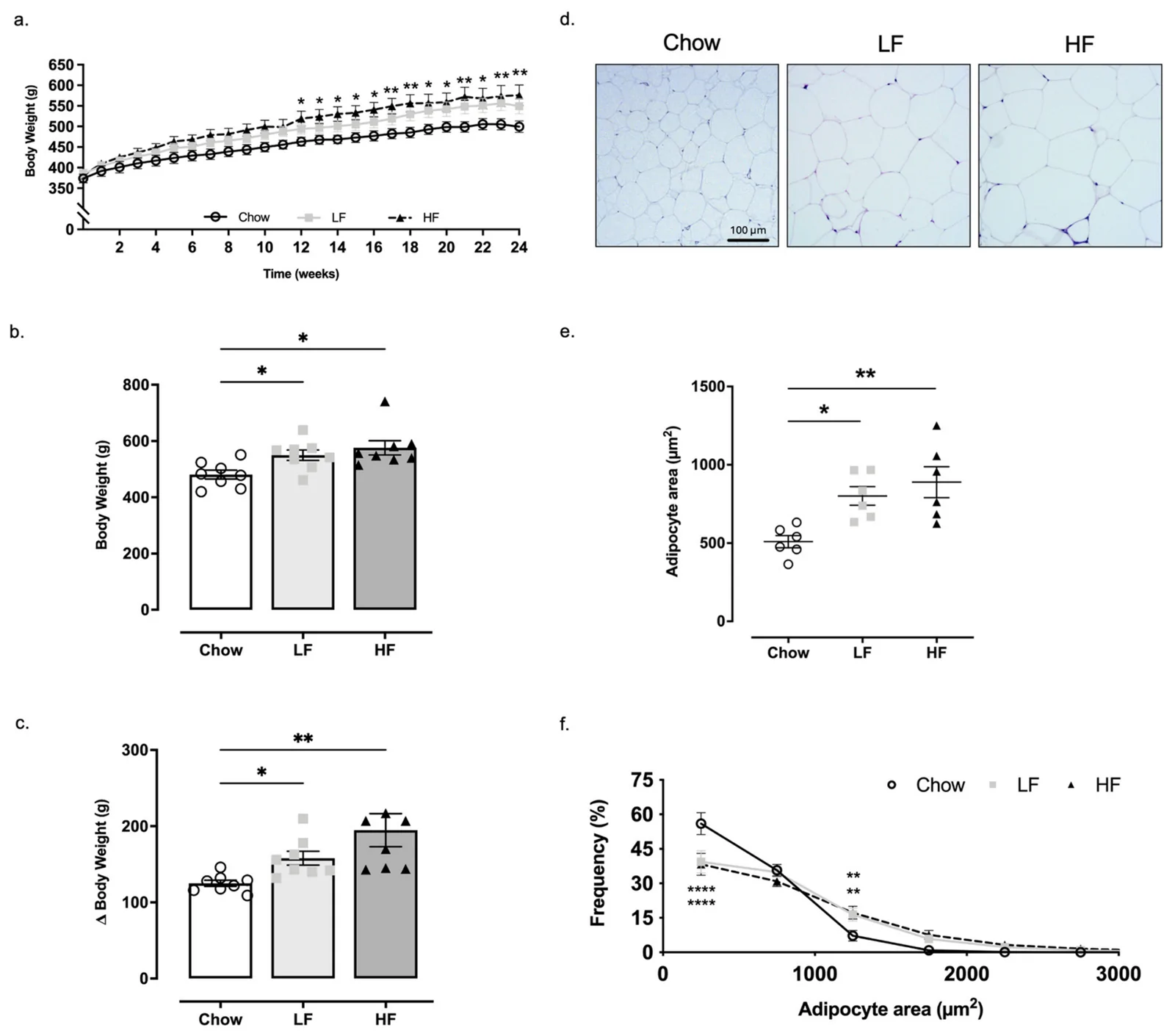
**Body weight evolution and adiposity.** (**a**) Body weight (g) recorded weekly throughout the 24-week protocol; (**b**) final body weight; (**c**) body weight gain (Δ body weight, g); (**d**) representative photomicrographs of H&E-stained epididymal adipose tissue (EAT); (**e**) adipocyte cross-sectional area (µm^2^); (**f**) adipocyte size-frequency distribution (% of adipocytes per area bin). Results are expressed as mean ± S.E.M of 6–8 animals per group. * *p* < 0.05, ** *p* < 0.01 and **** *p* < 0.0001 vs. Chow. Chow, non-purified whole-grain reference diet of similar energy density; LF, purified isocaloric low-fat diet; HF, purified high-fat, high-sucrose diet. Symbols represent individual animal values.

Body weight was monitored throughout the 24-week protocol (Figure 2a). Both processed diets promoted a progressive increase in body weight relative to Chow, reaching statistical significance from week 12 onwards in HF animals (*p* < 0.05 from weeks 12–17 and *p* < 0.01 thereafter). Notably, LF animals followed the HF trajectory closely and gained weight at a total gross energy intake that did not exceed, and was in fact lower than, that of Chow animals (*p* < 0.01; Table 4). Because metabolizable energy, energy expenditure and physical activity were not determined, this dissociation between recorded energy intake and weight gain is descriptive, and its basis remains undetermined. Final body weight and body weight gain are shown in Figure 2b,c.

Adiposity was assessed both as the mass of the epididymal fat depot and by morphometry of the same tissue. Epididymal WAT weight was 2.3-fold higher in LF-fed (16.28 ± 2.64 g) and 2.5-fold higher in HF-fed animals (17.55 ± 3.03 g) than in Chow-fed controls (7.10 ± 0.57 g; *p* < 0.01 and *p* < 0.001, respectively; Supplementary Table S2). It was subsequently evaluated by morphometric analysis of H&E-stained epididymal adipose tissue (Figure 2d). Adipocyte cross-sectional area was significantly increased in both LF (800.9 ± 59.2 µm^2^) and HF animals (889.0 ± 98.5 µm^2^) compared with Chow (509.5 ± 39.1 µm^2^; *p* < 0.05 and *p* < 0.01, respectively; Figure 2e). Accordingly, the adipocyte size-frequency distribution was shifted rightwards in both processed groups (Figure 2f), with a reduced proportion of small adipocytes (<250 µm^2^; *p* < 0.0001 for LF and HF vs. Chow) and a correspondingly higher proportion of large adipocytes (1250 µm^2^; *p* < 0.01 for both).

### 3.3. Effects of Low-Fat Diet on Glycemic and Insulinemic Profile

**Figure 3:**
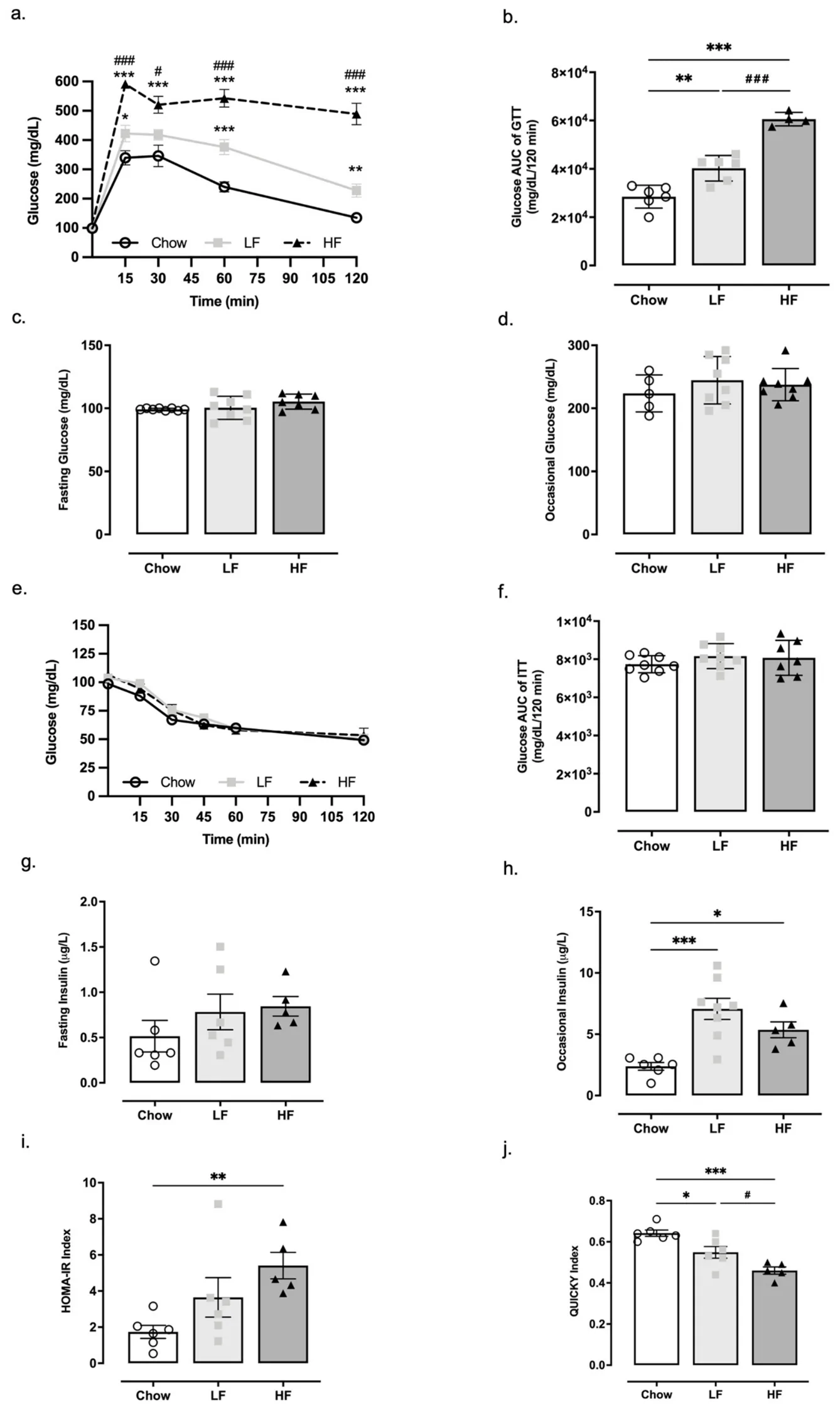
**Glycemic and insulinemic profile at week 24.** (**a**) Blood glucose levels (mg/dL) throughout the GTT; (**b**) AUC of the GTT curve (mg/dL × 120 min); (**c**) fasting glucose (mg/dL); (**d**) random-fed glucose (mg/dL); (**e**) blood glucose concentration throughout the ITT, expressed as absolute values (mg/dL); (**f**) AUC of the ITT curve (mg/dL × 120 min); (**g**) fasting insulin (μg/L); (**h**) random-fed insulin (μg/L); (**i**) HOMA-IR index; (**j**) QUICKI index. Results are expressed as mean ± S.E.M of 4–8 animals per group. * *p* < 0.05, ** *p* < 0.01 and *** *p* < 0.001 vs. Chow; # *p* < 0.05 and ### *p* < 0.001 vs. LF. AUC, area under the curve; GTT, glucose tolerance test; ITT, insulin tolerance test. Chow, non-purified whole-grain reference diet of similar energy density; LF, purified isocaloric low-fat diet; HF, purified high-fat, high-sucrose diet. Symbols represent individual animal values.

At week 24, both LF and HF animals displayed markedly higher glycemic excursions throughout the GTT (Figure 3a), translating into a significantly increased area under the curve (AUC) relative to Chow (*p* < 0.01 and *p* < 0.001, respectively), which was further amplified in HF compared with LF animals (*p* < 0.001; Figure 3b). Neither fasting nor random-fed glycemia differed among groups (Figure 3c,d). Peripheral insulin sensitivity was preserved under both regimens: the glucose decay curve during the ITT was comparable in Chow, LF and HF animals (Figure 3e), with no differences in the corresponding AUC (Figure 3f). Fasting insulin showed a non-significant trend towards higher values in LF and HF animals (Figure 3g), whereas random-fed insulin was significantly elevated in both LF (*p* < 0.001) and HF animals (*p* < 0.05) versus Chow (Figure 3h), consistent with a compensatory hyperinsulinemic response. The two fasting-based indices derived from these same measurements, and therefore not independent of them, reached significance where their individual components did not: HOMA-IR was increased in HF animals (*p* < 0.01 vs. Chow; Figure 3i), and QUICKI was progressively reduced in LF (*p* < 0.05) and HF animals (*p* < 0.001 vs. Chow; *p* < 0.05 HF vs. LF; Figure 3j). The divergence between the two indices follows from their construction: HOMA-IR is the product of untransformed values and is therefore more sensitive, at this sample size, to the animals with the highest insulin concentrations, whereas the logarithmic transformation in QUICKI normalizes the skewed distribution of insulin [52,53]. Together, these data indicate reduced fasting insulin sensitivity with preserved peripheral insulin action.

Overall, these findings indicate that an isocaloric, low-fat but poor-carbohydrate-quality diet was associated with a glycemic phenotype resembling that observed under hypercaloric high-fat feeding.

### 3.4. Effects of Low-Fat Diet on Gut Microbiota Composition and Gut-Derived Microbial Metabolites

**Figure 4:**
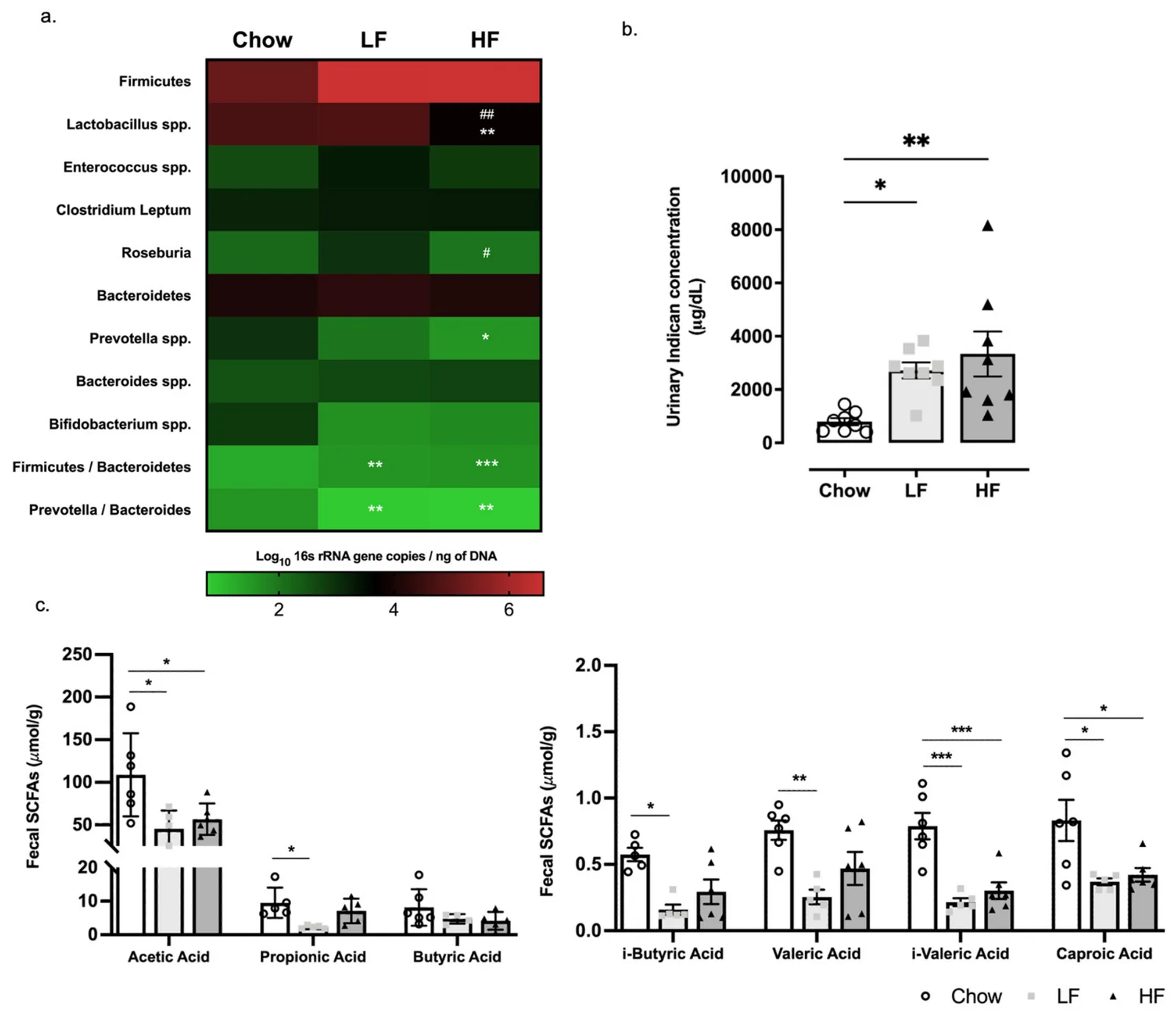
**Composition of gut microbiota and gut-derived microbial metabolites.** (**a**) Heat map of gut bacterial groups quantified by RT-PCR (Log_10_ 16S rRNA gene copies/ng of DNA), including the *Firmicutes*/*Bacteroidetes* and *Prevotella*/*Bacteroides* ratios; (**b**) urinary indican (Indoxyl-3-sulfate) concentration (μg/dL); (**c**) fecal short-chain fatty acids content (μmol/g). Results are expressed as mean ± S.E.M of 4–8 animals per group. * *p* < 0.05, ** *p* < 0.01 and *** *p* < 0.001 vs. Chow; # *p* < 0.05 and ## *p* < 0.01 vs. LF. Chow, non-purified whole-grain reference diet of similar energy density; LF, purified isocaloric low-fat diet; HF, purified high-fat, high-sucrose diet. Symbols represent individual animal values.

Given the poorer carbohydrate quality of the processed diets, we next examined whether this was reflected at the intestinal level by quantifying the main gut bacterial groups by RT-PCR (Figure 4a). HF-fed animals displayed the most pronounced compositional changes, with depletion of *Lactobacillus* spp. (*p* < 0.01 vs. Chow; *p* < 0.01 vs. LF), *Roseburia* spp. (*p* < 0.05 vs. LF) and *Prevotella* spp. (*p* < 0.05 vs. Chow). Importantly, both compositional ratios were altered in both processed groups: the *Firmicutes*/*Bacteroidetes* (F/B) ratio was significantly increased in LF and HF animals (*p* < 0.01 and *p* < 0.001 vs. Chow, respectively), whereas the *Prevotella*/*Bacteroides* ratio was decreased in both (*p* < 0.01 vs. Chow).

We next assessed the functional relevance of these microbial shifts by quantifying gut-derived bacterial metabolites, namely fecal short-chain fatty acids (SCFAs) and branched SCFAs, the end-products of bacterial saccharolytic and proteolytic fermentation; and urinary indican (indoxyl-3-sulfate, I3S), a tryptophan-derived uremic toxin generated by bacterial proteolytic fermentation (Figure 4b,c). Urinary I3S was elevated in both LF- (*p* < 0.05 vs. Chow) and HF-fed animals (*p* < 0.01 vs. Chow) and was mirrored by both SCFA and BSCFA depletion under both processed regimens. SCFA and BSCFA depletion was, however, most extensive under the LF diet: acetate (C2) was reduced in both LF and HF animals (*p* < 0.05 vs. Chow), whereas propionate (C3; *p* < 0.05), isobutyrate (i-C4; *p* < 0.05) and valerate (C5; *p* < 0.01) decreased only in LF animals; isovalerate (i-C5; *p* < 0.001) and caproate (C6; *p* < 0.05) were reduced in both groups, while butyrate (C4) did not differ significantly among groups. Collectively, this signature—an expanded F/B ratio, contraction of fiber-fermenting and *Prevotella*-type taxa, SCFA depletion and increased proteolytic metabolite output—was observed under the LF diet to an extent at least comparable to that of the HF diet despite its equivalent carbohydrate content.

### 3.5. Effects of Low-Fat Diet on Lipid Profile and Hepatic Steatosis

The serum lipid profile is presented in Table 5. Serum triglycerides did not differ significantly from Chow in either processed groups, although HF animals presented lower circulating triglyceride levels than LF animals (*p* < 0.05). LDL-cholesterol was significantly increased in both LF (*p* < 0.05) and HF animals (*p* < 0.01) relative to Chow, whereas HDL-cholesterol and total-cholesterol was comparable across all groups.

**Figure 5:**
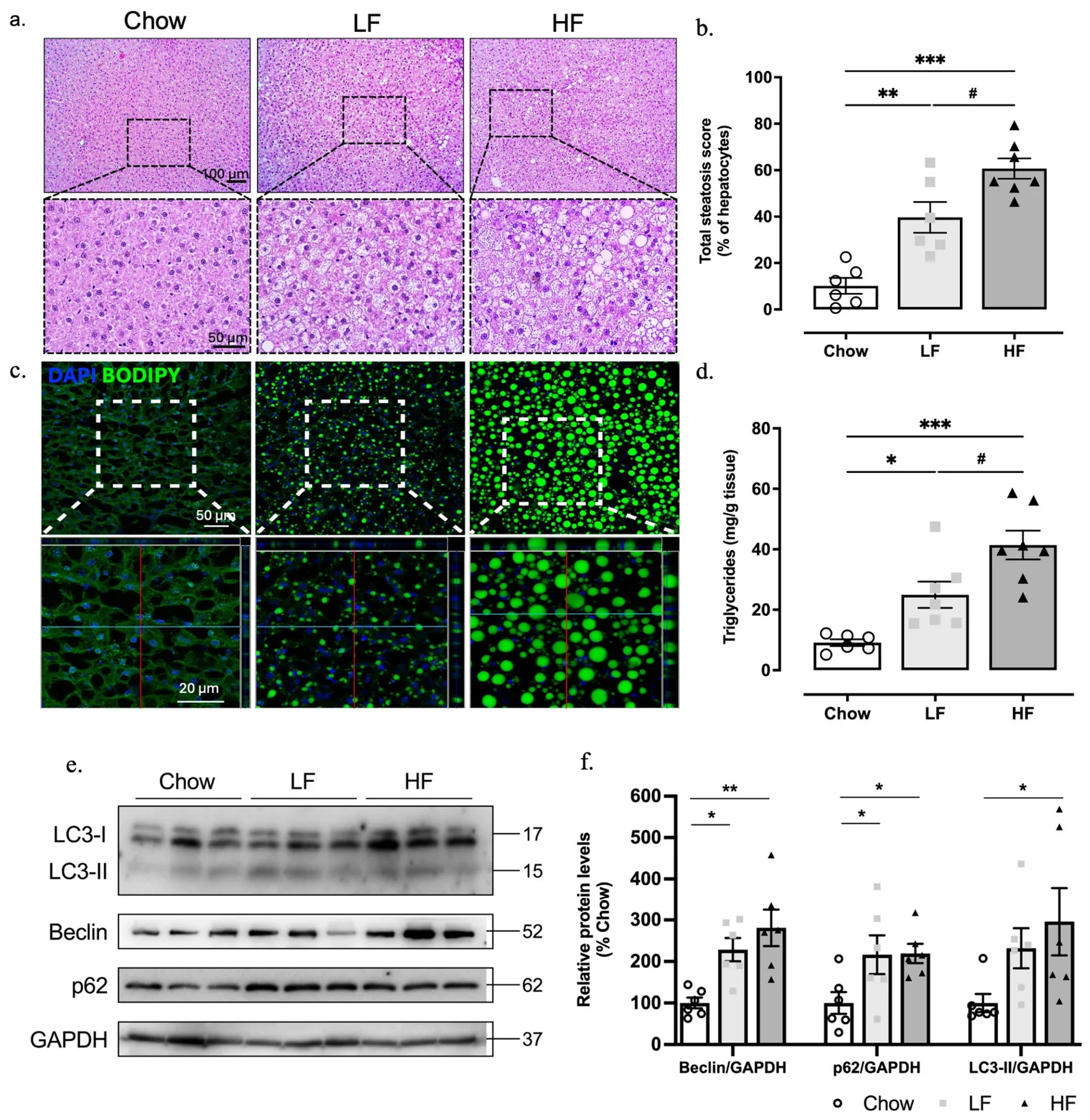
**Hepatic lipid profile.** (**a**) Representative photomicrographs of H&E-stained liver samples; (**b**) total steatosis score expressed as percentage of hepatocytes; (**c**) representative confocal images of hepatic neutral lipid deposition stained with Bodipy (green) and nuclei counterstained with DAPI (blue); (**d**) hepatic triglyceride levels (mg/g tissue); (**e**) representative Western blot of LC3-I, LC3-II, Beclin-1, p62 and GAPDH protein levels, expressed as % of Chow; (**f**) semi-quantitative analysis of LC3-I, LC3-II, Beclin-1, p62 and GAPDH protein levels (% Chow). Results are expressed as mean ± S.E.M of 4–8 animals per group. * *p* < 0.05, ** *p* < 0.01 and *** *p* < 0.001 vs. Chow; # *p* < 0.05 vs. LF. Chow, non-purified whole-grain reference diet of similar energy density; LF, purified isocaloric low-fat diet; HF, purified high-fat, high-sucrose diet. Symbols represent individual animal values.

Hepatic lipid deposition was next evaluated (Figure 5). H&E staining revealed accumulation of intracytoplasmic lipid droplets under both refined diets (Figure 5a), and the total steatosis score, expressed as the percentage of affected hepatocytes, was significantly increased in both LF (*p* < 0.01) and HF animals (*p* < 0.001) relative to Chow, with a further increment in HF versus LF animals (*p* < 0.05; Figure 5b). Confocal microscopy of BODIPY-stained sections showed a higher neutral lipid content, together with an increased number and size of lipid droplets in LF livers, an effect that was further magnified upon exposure to the HF diet (Figure 5c). Hepatic triglyceride quantification corroborated these findings, with increased content in both the LF (*p* < 0.05) and HF groups (*p* < 0.001 vs. Chow; *p* < 0.05 HF vs. LF; Figure 5d). Liver weight did not differ between groups (16.26 ± 0.75, 16.09 ± 0.99 and 14.82 ± 0.93 g in Chow-, LF- and HF-fed animals, respectively; Supplementary Table S2), indicating that the hepatic lipid accumulation occurred in the absence of hepatomegaly. In addition, serum ALT and AST did not differ between groups (22.13 ± 0.85, 23.75 ± 2.11 and 25.14 ± 1.22 U/L, and 66.50 ± 3.86, 70.50 ± 2.81 and 72.63 ± 10.75 U/L in Chow-, LF- and HF-fed animals, respectively; Supplementary Table S3), indicating hepatic lipid accumulation without overt hepatocellular injury.

Given that hepatic lipid handling is closely linked to autophagic flux, we next assessed the expression of autophagy markers by Western blot (Figure 5e,f). Beclin-1, which is required for autophagosome nucleation, was increased in LF (*p* < 0.05) and HF animals (*p* < 0.01) relative to Chow. Levels of p62, a selective autophagy receptor that is itself degraded within autolysosomes and therefore accumulates when autophagic flux is impaired, were likewise elevated in both groups (*p* < 0.05 vs. Chow), as was LC3-II, which reached statistical significance in HF-fed animals (*p* < 0.05 vs. Chow).

### 3.6. Effects of Low-Fat Diet on Kidney Structure and Function

Kidney weight did not differ between groups (2.93 ± 0.03, 2.93 ± 0.10 and 2.62 ± 0.10 g in Chow-, LF- and HF-fed animals, respectively; Supplementary Table S2). Renal function was assessed through the analysis of serum and urinary parameters (Table 6). The 24 h urine volume was significantly reduced only in HF animals (*p* < 0.05 vs. Chow), concordant with their lower fluid intake (*p* < 0.001). Serum creatinine was comparable across groups, whereas creatinine clearance was significantly reduced only in the LF group (74.740 ± 9.850 vs. 111.583 ± 6.889 mL/h in Chow; *p* < 0.05). Serum and urinary albumin, the albumin-to-creatinine ratio (ACR) and serum electrolytes did not differ significantly among groups. While comparable fasting glucose levels, measured in conscious animals, were observed across all groups, only LF animals developed glycosuria: urinary glucose was more than three-fold higher in the LF group than in Chow (*p* < 0.01), as well as than in the HF group (*p* < 0.001 vs. LF), with a parallel increase in glucose clearance restricted to the LF regimen (*p* < 0.05 vs. Chow; *p* < 0.01 HF vs. LF).

**Figure 6:**
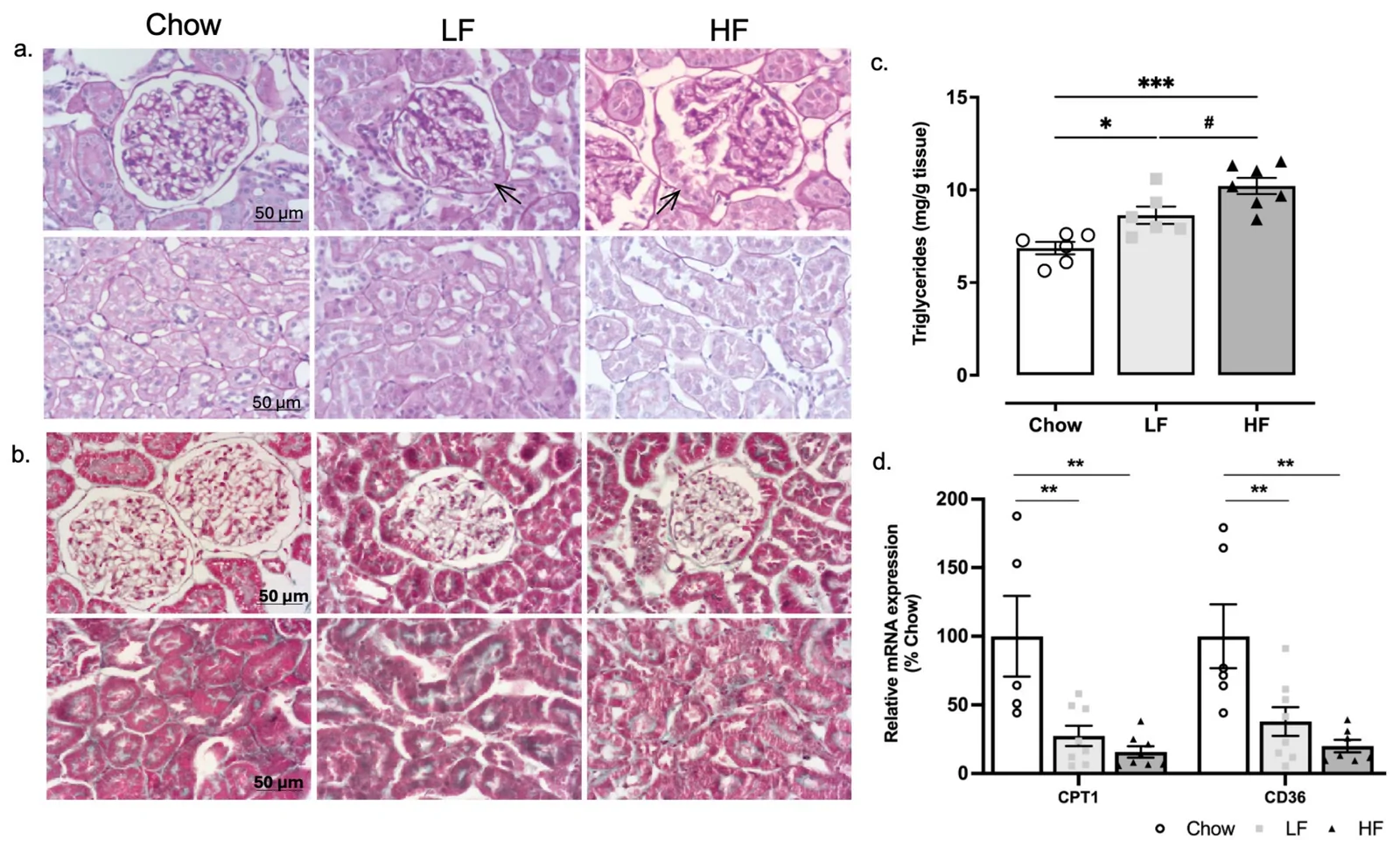
**Kidney histomorphology and renal lipidosis.** (**a**) Representative photomicrographs of PAS-stained kidney sections (upper row, glomeruli; lower row, tubules); arrows indicate crescent-like structures; (**b**) representative photomicrographs of Gomori trichrome-stained kidney sections (**upper** row, glomeruli; **lower** row, tubules); (**c**) renal triglyceride content (mg/g tissue); (**d**) relative renal Cpt1a and Cd36 mRNA expression (% of Chow); results are expressed as mean ± SEM of 4–8 animals per group. * *p* < 0.05, ** *p* < 0.01 and *** *p* < 0.001 vs. Chow, # *p* < 0.05 vs. LF. Cd36, fatty acid translocase; Cpt1a, carnitine palmitoyltransferase 1; PAS, periodic acid–Schiff. Chow, non-purified whole-grain reference diet of similar energy density; LF, purified isocaloric low-fat diet; HF, purified high-fat, high-sucrose diet. Symbols represent individual animal values.

Kidney histomorphology was assessed by PAS and Gomori trichrome staining (Figure 6a,b). Glomerular analysis revealed mild mesangial matrix expansion and structures resembling crescent-like lesions [41] in both the LF and HF groups, with no evident signs of glomerular hypertrophy or glomerulosclerosis. The tubular compartment displayed unremarkable histological features under both regimens, and no collagen deposition was detected by Gomori staining, with no evidence of fibrotic remodeling at this stage. Nevertheless, renal triglyceride content was significantly increased under both refined dietary regimens (*p* < 0.05 for LF and *p* < 0.001 for HF vs. Chow; Figure 6c). Moreover, both refined diets downregulated the gene expression of *Cpt1a*, encoding carnitine palmitoyltransferase 1, the rate-limiting enzyme controlling mitochondrial long-chain fatty acid uptake for β-oxidation [54]; and of *Cd36*, encoding the principal fatty acid translocase mediating long-chain fatty acid uptake across the plasma membrane [55] (*p* < 0.01 vs. Chow for both; Figure 6d).

**Figure 7:**
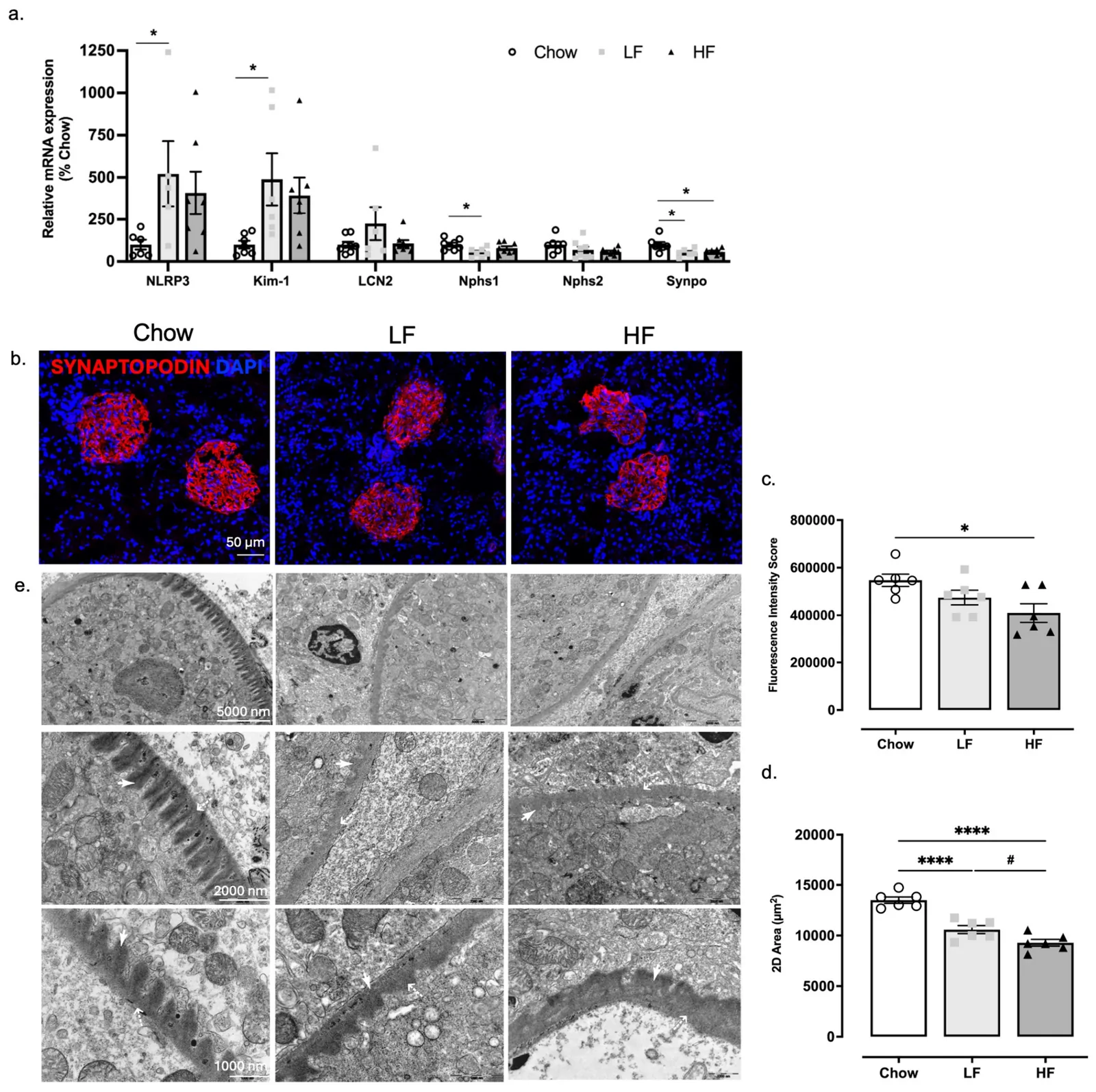
**Renal injury markers and podocyte integrity.** (**a**) Relative renal mRNA expression of injury markers (Nlrp3, KIM-1, Lcn2) and podocyte structural proteins (*Nphs1, Nphs2, Synpo*), expressed as % Chow; (**b**) representative confocal images of synaptopodin immunoreactivity (red) with nuclei counterstained with DAPI in blue; (**c**) synaptopodin fluorescence intensity score; (**d**) synaptopodin 2D-immunostained area (µm^2^); (**e**) representative transmission electron micrographs of kidney sections; ➛ highlights glomerular basement membrane and ⇢ highlights podocyte foot processes. Ultrastructural findings were assessed qualitatively. Results are expressed as mean ± S.E.M of 4–8 animals per group. * *p* < 0.05 and **** *p* < 0.0001 vs. Chow; # *p* < 0.05 vs. LF. KIM-1, kidney injury molecule-1; *Lcn2*, neutrophil gelatinase-associated lipocalin; *Nlrp3*, NLR family pyrin domain containing 3; *Nphs1*, nephrin; *Nphs2*, podocin; *Synpo*, synaptopodin. Chow, non-purified whole-grain reference diet of similar energy density; LF, purified isocaloric low-fat diet; HF, purified high-fat, high-sucrose diet. Symbols represent individual animal values.

Renal expression of injury-related genes was subsequently evaluated (Figure 7a). In LF-fed animals, there was an upregulation of *Nlrp3* and kidney injury molecule-1 (KIM-1) (*p* < 0.05 vs. Chow), with a non-significant increase in HF-fed animals; in the multiple-comparison analysis, in which all groups were compared, LF and HF did not differ from each other. By contrast, *Lcn2* (neutrophil gelatinase-associated lipocalin, NGAL) expression was not significantly altered. In addition, both dietary regimens were associated with podocyte-related structural and molecular alterations, evidenced by the downregulation of *Nphs1* and *Synpo* gene expression (*p* < 0.05 vs. Chow; Figure 7a), which encode nephrin and synaptopodin, respectively, the structural backbone of the slit diaphragm and the actin-associated protein that preserves foot process cytoskeletal architecture, whereas *Nphs2* (podocin) expression was preserved. Synaptopodin immunoreactivity corroborated these findings (Figure 7b), with a decreased fluorescence intensity score in HF animals (*p* < 0.05 vs. Chow; Figure 7c) and a reduced 2D-immunostained area in both LF and HF animals (*p* < 0.0001 vs. Chow), which was further reduced in HF compared with LF animals (*p* < 0.05; Figure 7d). Ultrastructural analysis by TEM showed thickening of the glomerular basement membrane together with irregular podocyte foot process dimensions and spacing in both LF- and HF-fed animals (Figure 7e).

## 4. Discussion

The central finding of the present study is that a purified diet formulated to carry a low-fat claim was not metabolically benign: it was associated with the same spectrum of alterations across the gut–liver–kidney axis as its ingredient-matched high-fat, high-sucrose counterpart. Over 24 weeks, LF-fed animals gained weight and developed adipocyte hypertrophy, impaired glucose tolerance accompanied by compensatory hyperinsulinemia, and gut dysbiosis characterized by a global reduction in colonic substrate availability. These metabolic alterations were accompanied by hepatic steatosis and renal lipidosis with early functional and ultrastructural abnormalities, evidenced by reduced creatinine clearance and structural podocyte abnormalities.

The LF and Chow diets differ in several respects beyond their carbohydrate fraction, each a candidate contributor to the phenotype. Low-fat provided a greater proportion of lipid-derived energy than Chow (13.0% vs. 8.6%, i.e., 1.5-fold), supplied as anhydrous milkfat with soybean oil rather than as the lipid fraction of whole grain, so the diets differ in fatty acid saturation as well as in fat quantity, both of which bear on hepatic lipid handling. The LF diet also provided a lower contribution of protein-derived energy (19.1% vs. 23.5%) and, because food intake was lower, a 26% lower absolute protein intake (80.33 ± 2.28 vs. 108.14 ± 1.28 kcal/week; *p* < 0.001; Table 4), which means that reduced protein availability cannot be excluded—including at the colonic level, where less undigested protein reaching the large bowel would itself lower the production of branched short-chain fatty acids. The purified diets further lack the phytate, phytochemicals and grain-derived micronutrients of the Chow matrix and supply fiber exclusively as poorly fermentable cellulose. None of these dimensions was varied independently, and the present design cannot apportion the phenotype among them.

What the design does establish is that LF and Chow were isocaloric and delivered an identical proportion of carbohydrate-derived energy (67.9%), so that the difference within the carbohydrate fraction is one of quality rather than quantity. Expressed as the modified CH:DF:FS ratio, only Chow (10.0:2.7:0.09) satisfies both components of the stringent 10:1:1 criterion; both refined formulations fall outside it, each providing less than 1 g of dietary fiber and more than 1 g of free sugars per 10 g of total carbohydrate (LF, 10.0:0.6:1.7; HF, 10.0:0.8:6.8) [7,8]. This pattern closely resembles that reported for commercially reformulated low-fat products, which tend to contain more sugar and less fiber than their full-fat counterparts [4,5]. The model may therefore have translational relevance to the low-fat food category, as it may recapitulate the reformulation trade-off inherent to these products and provide a framework for investigating how their chronic consumption may affect long-term metabolic health.

Low-fat animals gained weight along a trajectory similar to that of high-fat animals despite consuming fewer calories than Chow-fed controls (*p* < 0.01), and their epididymal depot more than doubled in mass. This expansion was predominantly hypertrophic: the 2.3-fold increase in depot mass in LF-fed animals is largely accounted for by the larger mean adipocyte cross-sectional area. Consistent with this, the size–frequency distribution showed no increase in the proportion of small adipocytes, which would be expected if new cells were being recruited. The LF diet was associated with marked glucose intolerance and elevated insulin levels, whereas fasting glycemia, the ITT decay curve, and its AUC remained unaltered. Fasting indices are dominated by hepatic insulin sensitivity, since fasting glycemia is set largely by hepatic glucose output, whereas an insulin tolerance test with exogenous insulin reports predominantly on peripheral glucose disposal [52]. The combination observed here—reduced QUICKI with an unaltered ITT decay curve and AUC—is therefore most consistent with reduced hepatic insulin sensitivity alongside preserved peripheral insulin action, an early stage of the metabolic trajectory in which normoglycemia is maintained at the cost of a higher insulin output [56]. A non-exclusive alternative should be acknowledged: approximately half of secreted insulin is extracted by the liver on first pass. Since hepatic steatosis reduces insulin extraction, part of the elevation in circulating insulin may reflect diminished hepatic insulin clearance rather than increased β-cell output [57]. This combination of findings may reflect an early stage of the metabolic disease trajectory, in which peripheral insulin sensitivity remains sufficient to clear an exogenous insulin bolus, while normoglycemia is maintained at the cost of a higher insulin output [56].

The microbial phenotype provides an integrated functional readout of the consequences of carbohydrate quality. Both processed diets increased the *Firmicutes*/*Bacteroidetes* (F/B) ratio (*p* < 0.01 and *p* < 0.001 vs. Chow for LF and HF, respectively) and reduced the *Prevotella*/*Bacteroides* ratio (*p* < 0.01 for both), the latter being an informative index of dietary fiber intake [58]. The high-fat diet additionally depleted *Lactobacillus* spp., *Roseburia* spp., and *Prevotella* spp. In functional terms, both dietary regimens were associated with a marked reduction in fecal SCFAs, encompassing acetate, propionate, isobutyrate, valerate, isovalerate, and caproate, with the LF diet producing the more extensive reduction in fermentation products. Although fecal SCFA concentrations do not directly quantify total microbial production, their reduction is consistent with diminished availability of fermentation-derived metabolites within the colonic environment. These changes may reflect differences in the availability of fermentable substrates to the colonic microbiota. Rapidly digestible starch, maltodextrin, and sucrose are hydrolyzed and absorbed predominantly in the proximal small intestine and are therefore unlikely to reach the colon in substantial amounts, whereas dietary fiber and resistant starch are more readily available for colonic fermentation [59,60]. From this perspective, the LF diet delivers a substantial carbohydrate load to the host while providing relatively little fermentable substrate to the colonic microbiota, potentially creating a state of colonic substrate deprivation [32,61]. Taken together, the combined signature of altered microbial composition, depletion of taxa associated with fiber fermentation, reduced fecal SCFA and BSCFA levels, and increased urinary secretion of indole-derived metabolites is consistent with a dysbiotic profile featured by a decrease in total fermentative output.

Given the reported consequences of altered microbial fermentative output for systemic physiology and inter-organ crosstalk, we next evaluated hepatic and renal parameters relevant to both structural integrity and function [17,28,62]. Both processed diets induced hepatic lipid accumulation, supported by three independent readouts (steatosis score, BODIPY-stained neutral lipid and tissue triglyceride content), with the most pronounced effects observed under high-fat feeding. Nevertheless, the development of steatosis in the LF group, despite deriving only 13% of its energy from fat and consuming fewer total calories than Chow-fed controls, argues against equating a low-fat designation with hepatoprotection. One possible explanation, consistent with the dietary composition, is that the carbohydrates replacing dietary fat may have favored hepatic de novo lipogenesis, potentially amplified by the accompanying hyperinsulinemia. The altered microbial environment, characterized by reduced SCFA availability and increased production of indole-derived metabolites, may represent an additional component of this metabolic context. Hepatic autophagy-related markers provided further evidence of altered intracellular lipid-handling pathways. Beclin-1, p62, and LC3-II were increased, and the concomitant accumulation of LC3-II and its cargo receptor p62 has been interpreted in other experimental settings as being more consistent with impaired lysosomal degradation than with increased autophagic flux [63], a pattern previously described in steatotic livers of diet-induced obese rodents [64]. Because lipophagy contributes to lipid-droplet clearance, impaired degradation could plausibly favor persistence of hepatic lipid accumulation. Nevertheless, autophagic flux was not directly measured in the present study; therefore, the observed changes in Beclin-1, p62, and LC3-II should be interpreted as evidence of altered autophagy-related signaling rather than definitive proof of impaired autophagic flux.

Renal lipid handling was also altered upon LF feeding. Renal triglyceride elevation paralleled a downregulation of Cpt1a and Cd36 gene transcription, a pattern consistent with reduced capacity for fatty-acid oxidation and uptake, respectively. Such alterations may represent an adaptive response to renal lipid accumulation and have been associated with fibrotic progression in experimental models [54,55,65,66]. Notably, only LF-fed animals developed glycosuria, at comparable fasting glycemia as well as reduced creatinine clearance. Normoglycemic glycosuria in the setting of a preserved filtered glucose load is more consistent with impaired proximal tubular reabsorption than with hyperglycemia-driven glucose spillover [67,68] and was accompanied by a transcriptional profile consistent with proximal tubular injury. KIM-1, a transmembrane glycoprotein that is virtually undetectable in the healthy kidney but markedly induced in dedifferentiated proximal tubular epithelial cells, is regarded as one of the earliest and most sensitive biomarkers of tubular injury [69,70]. Nlrp3, in turn, encodes the sensor component of the NLRP3 inflammasome, a cytosolic multiprotein complex that can be activated by metabolic danger signals, including lipid overload, hyperglycemia, and reactive oxygen species, leading to caspase-1-dependent maturation of IL-1β and IL-18 and thereby contributing to sterile renal inflammation [71,72]. The concurrent upregulation of KIM-1 and Nlrp3, reaching statistical significance only in the LF group, therefore suggests early proximal tubular involvement. The reduction in creatinine clearance also admits several explanations. A contribution of altered creatinine generation is unlikely: a clearance computed from simultaneous urine and plasma concentrations is largely independent of the generation rate, serum creatinine did not differ between groups, and LF-fed animals were heavier than controls. Renal hemodynamics were not assessed, and a contribution of reduced renal blood flow cannot be excluded. A tubular contribution is, however, supported by the concurrent induction of KIM-1 and by the fact that a substantial fraction of the renal clearance of creatinine is accounted for by active proximal tubular secretion rather than by filtration (PMID: 37977812) [73]. Creatinine clearance was also lower in HF-fed animals) without reaching significance, so the difference between the two refined diets in this parameter is one of statistical resolution. Still, proximal tubular injury remains a hypothesis rather than a demonstrated mechanism: future studies should address filtered glucose load, the fractional excretion of glucose and/or renal SGLT2 expression. Evidence of glomerular structural alterations was also present in both refined-diet groups. Nphs1 and Synpo gene expression were reduced, synaptopodin immunoreactivity and stained area were decreased, and transmission electron microscopy revealed glomerular basement membrane thickening together with irregular foot-process architecture. This pattern resembles the podocytopathy described in obesity-related glomerulopathy, in which podocyte stress, hypertrophy, and eventual loss are thought to precede overt albuminuria [74,75,76,77]. This interpretation is further supported by evidence that podocyte density may predict long-term outcome in obesity-related glomerulopathy before conventional functional markers become abnormal [77].

Taken together, the present findings highlight that the metabolic consequences of a low-fat diet depend not only on how much fat is removed but also on what replaces it. In the present model, an isocaloric low-fat formulation characterized by fiber depletion, free-sugar enrichment, and a greater contribution from rapidly digestible starch was associated with alterations in the gut microbial community and reduced fecal SCFA and BSCFA levels accompanied by increased urinary I3S. These changes occurred alongside hepatic steatosis and altered autophagy-related markers, suggesting that the quality of the replacement carbohydrates may be relevant to hepatic lipid handling. In the kidney, the same dietary context was associated with renal lipid accumulation, normoglycemic glycosuria, reduced creatinine clearance, proximal tubular alterations, and structural podocyte abnormalities. Although the present study cannot establish direct causal links between individual dietary components, microbial metabolites, and organ-specific injury, the concordance of these findings across metabolic, microbial, hepatic, and renal endpoints provides a coherent picture of concurrent alterations across the three organs. The interconnected nature of these alterations is consistent with the concept of gut–liver–kidney crosstalk [17,28,62] and may provide a biological context for the epidemiological association between MASLD and increased risk of incident CKD [16,78,79]. More broadly, the findings caution against interpreting dietary fat reduction in isolation from the nutritional characteristics of the foods used to achieve it. A reduction in dietary fat does not necessarily translate into metabolic benefit when accompanied by deterioration in carbohydrate quality. Within the limitations inherent to an experimental animal model, the present data therefore support the concept that the metabolic effects of low-fat formulations are shaped by the broader nutrient matrix in which fat reduction occurs, with potential implications for the long-term metabolic health consequences of highly processed low-fat foods.

## 5. Study Strengths and Limitations

A major strength of this study is the comprehensiveness of the phenotyping strategy, which integrated metabolic, microbial, hepatic, and renal endpoints, ranging from microbial composition and fecal fermentation products to hepatic lipid accumulation, renal functional parameters, and glomerular ultrastructure. This multidimensional approach enabled the identification of convergent alterations across the gut–liver–kidney axis rather than relying on isolated metabolic outcomes.

Several limitations should nevertheless be acknowledged. First, the CH:DF:FS ratio is a food-based descriptor developed for human diets; it is applied here to characterize the composition of the experimental diets and not as a validated predictor of metabolic responses in rodents. Second, the study did not include a purified diet matched to LF for dietary fiber and free sugars but formulated with slowly digestible starch. Such an experimental arm would have allowed the effects of starch digestibility to be isolated within an otherwise identical purified background and, when combined with a fiber or short-chain fatty acid repletion arm, could have helped determine whether colonic substrate deprivation is necessary for the phenotype described here. Moreover, the exclusive use of male rats, intended to limit biological heterogeneity within the experimental design, precludes the assessment of sex-specific responses and limits the generalizability of these findings. Because rats are a social species and were pair-housed as a welfare refinement, dietary treatment could only be allocated at the cage level; consequently, values obtained from cage mates cannot be considered strictly independent. In addition, urine and feces were collected in metabolic cages without a preceding acclimatization period, which may have introduced a transient stress component common to all three groups. Insulin was quantified without *C*-peptide, so increased secretion cannot be distinguished from reduced hepatic insulin clearance, and no islet morphometry was performed to document β-cell compensation.

Other methodological considerations should also be highlighted. Microbiota profiling was targeted rather than comprehensive and was performed by RT-PCR rather than 16S rRNA gene sequencing, limiting the resolution of community-level changes and precluding a broader assessment of microbial diversity and composition. The *Firmicutes*/*Bacteroidetes* ratio should likewise be interpreted with caution: it aggregates two phyla of considerable internal functional heterogeneity, its association with obesity and metabolic disease has proved inconsistent across studies, and its value as a summary index of dysbiosis has been questioned [80]. It is reported here as a descriptive compositional summary, and the functional interpretation rests on the fiber-fermenting taxa, the *Prevotella*/*Bacteroides* ratio and the fecal short-chain fatty acid concentrations. In addition, autophagic flux was not directly measured; therefore, changes in LC3-II, p62, and Beclin-1 cannot by themselves establish alterations in autophagic flux. Similarly, portal endotoxin concentrations, intestinal permeability, and circulating I3S were not assessed, preventing direct evaluation of intestinal barrier function and the systemic exposure to gut-derived microbial products. Finally, the absence of these measurements limits the extent to which mechanistic links between dietary composition, microbial alterations, and hepatic or renal injury can be inferred. Further studies incorporating comprehensive microbiome profiling, direct assessment of intestinal permeability and endotoxemia, and measurement of circulating microbial metabolites will be required to clarify these pathways.

Several constraints also apply to the renal phenotyping. Glomerular and tubular lesions were assessed descriptively, without a standardized scoring system, as well as ultrastructural findings, without morphometry of glomerular basement membrane thickness or foot-process width. Synaptopodin-immunostained area was not normalized to glomerular area, although no glomerular hypertrophy was apparent on histological examination. KIM-1 was measured at the transcript level only; protein and urinary KIM-1, which would have provided a functional correlate, were not determined. The absence of blood pressure measurements is of particular relevance to the renal findings since a hemodynamic contribution to the reduced creatinine clearance cannot be assessed.

## 6. Conclusions

In summary, over 24 weeks, a purified diet that would qualify for a low-fat claim was accompanied by the same spectrum of gut, liver and kidney alterations as its ingredient-matched high-fat, high-sucrose counterpart, at a gross energy intake that did not exceed that of a conventional whole-grain reference diet. Within the constraints of the experimental design, these findings identify refined low-fat formulations as an exposure that a fat-based nutritional claim does not describe. More broadly, they suggest that nutritional classifications based primarily on macronutrient thresholds may overlook the metabolic consequences of food reformulation, particularly when fat reduction is accompanied by depletion of dietary fiber and enrichment in free sugars. From a public health perspective, these findings underscore the need for nutrient-profiling and reformulation frameworks that move beyond fat thresholds to explicitly consider carbohydrate quality.

## Figures and Tables

**Table 1:** Primer sequences and real-time PCR conditions used for gut microbiota analysis.

Target Group	Primer Sequence (5′-3′)	Genomic DNA Standard	PCR Product Size (bp)	AT	Ref.
*Firmicutes*	ATG TGG TTT AAT TCG AAG CA AGC TGA CGA CAA CCA TGC AC	*Lactobacillus gasseri* ATCC 33323	126	45 °C	[43]
*Lactobacillus*	CAC CGC TAC ACA TGG AG AGC AGT AGG GAA TCT TCC A	*Lactobacillus gasseri* ATCC 33323	351	55 °C	[44]
*Enterococcus*	CCC TTA TTG TTA GTT GCC ATC ATT ACT CGT TGT ACT TCC CTT GT	*Enterococcus gilvus* ATCC BAA-350	144	50 °C	[45]
*Clostridium* *leptum*	GCA CAA GCA GTG GAG T CTT CCT CCG TTT TGT CAA	*Clostridium leptum* ATCC 29065	239	45 °C	[46]
*Roseburia*	TAC TGC ATT GGA AAC TGT CG CGG CAC CGA AGA GCA AT	*Roseburia hominis* A2-183	230	50 °C	[47]
*Bacteroidetes*	CAT GTG GTT TAA TTC GAT GAT AGC TGA CGA CAA CCA TGC AG	*Bacteroides vulgatus* ATCC 8482	126	45 °C	[43]
*Prevotella*	CAC RGT AAA CGA TGG ATG CC GGT CGG GTT GCA GAC C	*Prevotella nigrescens* ATCC 33563	513	50 °C	[48]
*Bacteroides*	ATA GCC TTT CGA AAG RAA GAT CCA GTA TCA ACT GCA ATT TTA	*Bacteroides vulgatus* ATCC 8482	495	45 °C	[48]
*Bifidobacterium*	CGC GTC YGG TGT GAA AG CCC CAC ATC CAG CAT CCA	*Bifidobacterium longum* subsp. *infantis* ATCC 15697	244	50 °C	[44]

AT, annealing temperature; bp, base pairs.

**Table 2:** Primer sequences and real-time PCR conditions used for host gene expression analysis.

Gene	Primer Sequence		Temp. (°C)
	Forward	Reverse	
β-actin	GGCACCACACTTTCTACAA	AGGTCTCAAACATGATCTGG	58
HPRT	ATGGGAGGCCATCACATTGT	ATGTAATCCAGCAGGTCAGCAA	60
Cpt1a	CCTACCACGGCTGGATGTTT	TACAACATGGGCTTCCGACC	56
NLRP3	CGGTGACCTTGTGTGTGCTT	TCATGTCCTGAGCCATGGAAG	60
Kim-1	ACTCCTGCAGACTGGAATGG	CAAAGCTCAGAGAGCCCATC	60
CD36	qRnoCED0007736 (Biorad catalog assay)		60
LCN2	qRnoCID0001706 (Biorad catalog assay)		60
Nphs1	qRnoCID0017962 (Biorad catalog assay)		60
Nphs2	qRnoCID0003013 (Biorad catalog assay)		60
Synpo	qRnoCID0002477 (Biorad catalog assay)		60

**Table 3:** Primary and secondary antibodies used in Western blot analysis.

Antibody	Reference	Source	Animal Origin	Blocking Solution	Solution	Dilution	Incubation Time
Anti-Beclin	Ab62557	Abcam	Rabbit	5% BSA in TBS-T	1% BSA in TBS-T	1:1000	24 h
Anti- SQSTM1/p62	5114S	Cell Signaling Technology	Rabbit	5% BSA in TBS-T	1% BSA in TBS-T	1:1000	24 h
Anti-LC3	PA1-16931	Thermo Fisher Scientific	Rabbit	5% BSA in TBS-T	1% BSA in TBS-T	1:1000	24 h
Anti-GAPDH	AB0049-200	Sicgen	Goat	5% BSA in TBS-T	1% BSA in TBS-T	1:5000	24 h
Anti-Rabbit	R-0572-050	Advansta	Goat	Same as respective antibodies		1:10,000	1 h
Anti-Goat	AB1011-1000	Sicgen	Goat			1:10,000	1 h

BSA: bovine serum albumin; GAPDH: glyceraldehyde 3-phosphate dehydrogenase; LC3: microtubule-associated protein 1 light chain 3; SQSTMI/p62: sequestosome-1/protein p62; TBS-T: tris-buffered saline with Tween-20.

**Table 4:** Beverage consumption, food intake and caloric intake. Data are expressed as mean ± SEM (n = 6–8 *per* group). * *p* < 0.05, ** *p* < 0.01 and *** *p* < 0.001 vs. Chow; ^##^ *p* < 0.01 and ^###^ *p* < 0.001 vs. LF.

Parameter	Chow	LF	HF
Food Intake (g/week)	146.09 ± 1.73	116.81 ± 3.31 ***	104.79 ± 4.24 ***
Beverage Intake (mL/week)	200.11 ± 2.17	187.94 ± 3.40	175.80 ± 4.82 ***
Calories from Carbohydrates (kcal/week)	312.47 ± 3.70	285.51 ± 8.10 *	200.43 ± 8.12 ***^, ###^
Calories from Lipids (kcal/week)	39.58 ± 0.47	54.66 ± 1.55 ***	219.65 ± 8.89 ***^, ###^
Calories from Proteins (kcal/week)	108.14 ± 1.28	80.33 ± 2.28 ***	72.40 ± 2.93 ***
∑ Total calories (kcal/week)	460.18 ± 5.45	420.51 ± 11.93 **	492.49 ± 19.93 ^##^

**Table 5:** Serum lipid profile. Data are expressed as mean ± SEM (n = 6–8 per group). * *p* < 0.05, ** *p* < 0.01 vs. Chow; ^#^ *p* < 0.05 vs. LF.

Parameter	Chow	LF	HF
Triglycerides (mg/dL)	219.50 ± 28.26	238.38 ± 21.79	147.43 ± 19.38 ^#^
LDL-c (mg/dL)	4.17 ± 0.48	7.75 ± 0.92 *	8.43 ± 0.61 **
HDL-c (mg/dL)	24.17 ± 1.49	29.25 ± 3.34	27.88 ± 2.20
Total Cholesterol (mg/dL)	73.00 ± 5.27	99.33 ± 14.44	77.88 ± 5.89

**Table 6:** Kidney function profile. Data are expressed as mean ± SEM (n = 6–8 per group). * *p* < 0.05, ** *p* < 0.01 vs. Chow; ^##^ *p* < 0.01 and ^###^ *p* < 0.001 vs. LF.

	Parameter	Chow	LF	HF
**Urine Output (24 h)**	Urine Volume (ml)	10.333 ± 0.616	9.000 ± 1.526	6.250 ± 0.453 *
**Creatinine (Cr)**	Serum (mg/dL)	0.636 ± 0.015	0.656 ± 0.017	0.639 ± 0.019
	Urine (mg/dL)	161.517 ± 10.384	149.767 ± 16.889	237.950 ± 13.149 **^, ##^
	Clearance (ml/h)	111.583 ± 6.889	74.740 ± 9.850 *	85.700 ± 7.985
	Clearance/BW (ml/h/g)	0.223 ± 0.011	0.140 ± 0.018 **	0.179 ± 0.015 *
**Albumin (A)**	Serum (mg/dL)	281.667 ± 4.773	300.000 ± 6.814	286.250 ± 4.978
	Urine (mg/dL)	0.564 ± 0.056	0.764 ± 0.097	0.838 ± 0.096
	A/Cr ratio (ACR)	3.933 ± 0.456	5.140 ± 0.498	3.463 ± 0.358
**Glucose**	Serum (mg/dL)	223.600 ± 13.102	244.500 ± 13.344	237.500 ± 9.034
	Urine (mg/dL)	24.167 ± 4.700	79.000 ± 16.508 **	23.750 ± 3.160 ^###^
	Clearance (ml/h)	0.043 ± 0.003	0.126 ± 0.038 *	0.025 ± 0.004 ^##^
**Serum Electrolytes**	Sodium (mmol/L)	140.875 ± 0.666	141.875 ± 1.457	141.500 ± 1.300
	Potassium (mmol/L)	6.312 ± 0.156	6.138 ± 0.222	6.188 ± 0.258
	Phosphorous (mg/dL)	6.025 ± 0.232	5.713 ± 0.238	5.988 ± 0.215

## Data Availability

The original contributions presented in this study are included in the article. Further inquiries can be directed to the corresponding author.

## Supplementary Materials

The following supporting information can be downloaded at https://www.mdpi.com/article/10.3390/nu18213718/s1, Table S1: Number of animals contributing to each analysis. Table S2. Liver, kidney, and white adipose tissue (WAT) weights. Table S3. Serum alanine aminotransferase (ALT) and aspartate aminotransferase (AST) levels.

## Abbreviations

The following abbreviations are used in this manuscript:

2D	Two-dimensional
ACR	Albumin-to-creatinine ratio
ANOVA	Analysis of variance
AOAC	Association of Official Analytical Chemists
ARRIVE	Animal Research: Reporting of In Vivo Experiments
AT	Annealing temperature
AUC	Area under the curve
BCA	Bicinchoninic acid
BODIPY	Boron-dipyrromethene
bp	Base pairs
BSA	Bovine serum albumin
BW	Body weight
cDNA	Complementary deoxyribonucleic acid
CD36/Cd36	Cluster of differentiation 36/fatty acid translocase
CH	Carbohydrate
CH:DF:FS	Carbohydrate-to-dietary fiber-to-free sugars ratio
CKD	Chronic kidney disease
Cpt1a	Carnitine palmitoyltransferase 1-a
DAPI	4′,6-Diamidino-2-phenylindole
DEPC	Diethyl pyrocarbonate
DF	Dietary fiber
DNA	Deoxyribonucleic acid
EAT	Epididymal adipose tissue
ECL	Enhanced chemiluminescence
EDTA	Ethylenediaminetetraacetic acid
ELISA	Enzyme-linked immunosorbent assay
F/B	Firmicutes-to-Bacteroidetes ratio
FBS	Fetal bovine serum
FS	Free sugars
GAPDH	Glyceraldehyde 3-phosphate dehydrogenase
GC	Gas chromatography
GTT	Glucose tolerance test
H&E	Hematoxylin and eosin
HDL-c	High-density lipoprotein cholesterol
HF	High-fat diet
HPLC	High-performance liquid chromatography
HPRT	Hypoxanthine-guanine phosphoribosyltransferase
I3S	Indoxyl-3-sulfate
IgG	Immunoglobulin G
IL-1β	Interleukin-1 beta
IL-18	Interleukin-18
ITT	Insulin tolerance test
KIM-1	Kidney injury molecule-1
LC3	Microtubule-associated protein 1 light chain 3
LCN2/Lcn2	Lipocalin-2
LDL-c	Low-density lipoprotein cholesterol
LF	Low-fat diet
MASLD	Metabolic dysfunction-associated steatotic liver disease
mRNA	Messenger ribonucleic acid
NGAL	Neutrophil gelatinase-associated lipocalin
NLRP3/Nlrp3	NLR family pyrin domain containing 3
Nphs1	Nephrin
Nphs2	Podocin
PAS	Periodic acid–Schiff
PBS	Phosphate-buffered saline
PCR	Polymerase chain reaction
PMSF	Phenylmethylsulfonyl fluoride
PVDF	Polyvinylidene fluoride
RI	Refractive index
RIPA	Radioimmunoprecipitation assay
RNA	Ribonucleic acid
ROI(s)	Region(s) of interest
rRNA	Ribosomal ribonucleic acid
RT-PCR	Real-time polymerase chain reaction
SCFA(s)	Short-chain fatty acid(s)
SDS	Sodium dodecyl sulfate
SDS-PAGE	Sodium dodecyl sulfate–polyacrylamide gel electrophoresis
SEM	Standard error of the mean
SQSTM1/p62	Sequestosome-1/protein p62
Synpo	Synaptopodin
TBS-T	Tris-buffered saline with Tween-20
TEM	Transmission electron microscopy
TGs	Triglycerides
WHO	World Health Organization
