# Supplementary material for: Low-Fat by Formulation, Not by Benefit: A Purified Low-Fat Diet Is Associated with Gut–Liver–Kidney Alterations in Rats
Source: Nutrients. 2026 Nov 9;18(21):3718. doi: 10.3390/nu18213718 (PMC13766120; doi:10.3390/nu18213718)
Supplement: Supplementary file 1 [file nutrients-18-03718-s001.zip › nutrients-4615305-supplementary.pdf]

Table S1. Number of animals contributing to each analysis.

Endpoint	Figure / Table	n Chow	n LF	n HF	Reason for departure from n = 8
Body weight (weekly) / final body weight/ body weight gain	Fig. 2a,b,c	8	8	8	Not applicable
Adipocyte cross-sectional area and size distribution	Fig. 2e,f	6	6	6	Endpoints performed in a predefined subset of animals because of the throughput of the technique
Food, beverage and caloric intake	Table 4	6	8	8	Technical failure of an individual measurement
GTT curve and AUC	Fig. 3a,b	6	6	4	Technical failure — injection; Exclusion of individual values identified as statistical outliers
Fasting glucose	Fig. 3c	8	8	7	Exclusion of individual values identified as statistical outliers
Occasional glucose	Fig. 3d	5	8	8	Exclusion of individual values identified as statistical outliers
ITT curve and AUC	Fig. 3e,f	8	8	7	Technical failure — injection
Fasting insulin	Fig. 3g	6	6	5	Technical failure of an individual measurement; Exclusion of individual values identified as statistical outliers
Occasional insulin	Fig. 3h	6	8	5	Technical failure of an individual measurement; Exclusion of individual values identified as statistical outliers
HOMA-IR and QUICKY index	Fig. 3i,j	6	6	5	Limited by the number of values of fasting insulin and fasting glucose.
Faecal microbiota (qPCR, 16S rRNA gene copies) - *Firmicutes*	Fig. 4a	6	4	6	Endpoint assessed in a subset of animals due to fecal sample availability; adequate fecal samples were not obtained from all animals.
Faecal microbiota (qPCR, 16S rRNA gene copies) - *Lactobacillus spp.*	Fig. 4a	5	4	6	Endpoint assessed in a subset of animals due to fecal sample availability; adequate fecal samples were not obtained from all animals; Exclusion of individual values identified as statistical outliers

Faecal microbiota (qPCR, 16S rRNA gene copies) - *Enterococcus spp.*	Fig. 4a	4	4	5	Endpoint assessed in a subset of animals due to fecal sample availability; adequate fecal samples were not obtained from all animals;<br>Exclusion of individual values identified as statistical outliers
Faecal microbiota (qPCR, 16S rRNA gene copies) - *Clostridium Leptum*	Fig. 4a	6	4	6	Endpoint assessed in a subset of animals due to fecal sample availability; adequate fecal samples were not obtained from all animals;
Faecal microbiota (qPCR, 16S rRNA gene copies) - *Roseburia*	Fig. 4a	6	4	6	Endpoint assessed in a subset of animals due to fecal sample availability; adequate fecal samples were not obtained from all animals;
Faecal microbiota (qPCR, 16S rRNA gene copies) - *Bacteroidetes*	Fig. 4a	6	4	6	Endpoint assessed in a subset of animals due to fecal sample availability; adequate fecal samples were not obtained from all animals;<br>Exclusion of individual values identified as statistical outliers
Faecal microbiota (qPCR, 16S rRNA gene copies) - *Prevotella spp.*	Fig. 4a	5	4	4	Endpoint assessed in a subset of animals due to fecal sample availability; adequate fecal samples were not obtained from all animals;<br>Exclusion of individual values identified as statistical outliers
Faecal microbiota (qPCR, 16S rRNA gene copies) - *Bacteroides spp.*	Fig. 4a	4	4	6	Endpoint assessed in a subset of animals due to fecal sample availability; adequate fecal samples were not obtained from all animals;<br>Exclusion of individual values identified as statistical outliers
Faecal microbiota (qPCR, 16S rRNA gene copies) - *Bifidobacterium spp.*	Fig. 4a	5	4	6	Endpoint assessed in a subset of animals due to fecal sample availability; adequate fecal samples were not obtained from all animals;<br>Exclusion of individual values identified as statistical outliers
Faecal microbiota (qPCR, 16S rRNA gene copies) - *Firmicutes / Bacteroidetes*	Fig. 4a	6	4	6	Endpoint assessed in a subset of animals due to fecal sample availability; adequate fecal samples were not obtained from all animals;
Faecal microbiota (qPCR, 16S rRNA gene copies) - *Prevotella / Bacteroides*	Fig. 4a	5	4	6	Endpoint assessed in a subset of animals due to fecal sample availability; adequate fecal samples were not obtained from all animals;<br>Exclusion of individual values identified as statistical outliers

Urinary Indican concentration	Fig. 4b	8	8	8	Not applicable
Faecal short-chain fatty acids – Acetic Acid	Fig. 4c	6	5	6	Endpoint assessed in a subset of animals due to fecal sample availability; adequate fecal samples were not obtained from all animals;
Faecal short-chain fatty acids – Propionic Acid	Fig. 4c	5	5	5	Endpoint assessed in a subset of animals due to fecal sample availability; adequate fecal samples were not obtained from all animals; Exclusion of individual values identified as statistical outliers
Faecal short-chain fatty acids – Butyric Acid	Fig. 4c	6	4	5	Endpoint assessed in a subset of animals due to fecal sample availability; adequate fecal samples were not obtained from all animals; Exclusion of individual values identified as statistical outliers
Faecal short-chain fatty acids – i-Butyric Acid	Fig. 4c	5	5	6	Endpoint assessed in a subset of animals due to fecal sample availability; adequate fecal samples were not obtained from all animals; Exclusion of individual values identified as statistical outliers
Faecal short-chain fatty acids – Valeric Acid	Fig. 4c	6	5	6	Endpoint assessed in a subset of animals due to fecal sample availability; adequate fecal samples were not obtained from all animals;
Faecal short-chain fatty acids – i-Valeric Acid	Fig. 4c	6	5	6	Endpoint assessed in a subset of animals due to fecal sample availability; adequate fecal samples were not obtained from all animals;
Faecal short-chain fatty acids – Caproic Acid	Fig. 4c	6	5	6	Endpoint assessed in a subset of animals due to fecal sample availability; adequate fecal samples were not obtained from all animals;
Serum lipid profile - Triglycerides	Table 5	6	8	7	Exclusion of individual values identified as statistical outliers
Serum lipid profile – LDL-c	Table 5	6	8	8	Exclusion of individual values identified as statistical outliers
Serum lipid profile – HDL-c	Table 5	6	8	8	Exclusion of individual values identified as statistical outliers
Serum lipid profile – Total Cholesterol	Table 5	6	6	8	Exclusion of individual values identified as statistical outliers
Hepatic steatosis score	Fig. 5b	6	6	7	Exclusion of individual values identified as statistical outliers
Hepatic triglyceride content	Fig. 5d	6	7	7	Exclusion of individual values identified as statistical outliers

Hepatic LC3-I/II, Beclin-1, p62 (Western blot)	Fig. 5e,f	6	6	6	Endpoints performed in a predefined subset of animals because of the throughput of the technique
Renal triglyceride content	Fig. 6c	6	6	7	Exclusion of individual values identified as statistical outliers
Renal Cpt1 mRNA	Fig. 6d	5	8	8	Exclusion of individual values identified as statistical outliers
Renal Cd36 mRNA	Fig. 6d	6	8	7	Exclusion of individual values identified as statistical outliers
Renal Nlrp3 mRNA	Fig. 7a	6	5	7	Exclusion of individual values identified as statistical outliers
Renal KIM-1 mRNA	Fig. 7a	7	6	7	Exclusion of individual values identified as statistical outliers
Renal Lcn2 mRNA	Fig. 7a	8	6	8	Exclusion of individual values identified as statistical outliers
Renal Nphs1 mRNA	Fig. 7a	8	7	8	Exclusion of individual values identified as statistical outliers
Renal Nphs2 mRNA	Fig. 7a	7	8	8	Exclusion of individual values identified as statistical outliers
Renal Synpo mRNA	Fig. 7a	7	6	7	Exclusion of individual values identified as statistical outliers
Synaptopodin immunoreactivity and stained area	Fig. 7c,d	6	6	6	Endpoints performed in a predefined subset of animals because of the throughput of the technique
Serum and 24-h urine renal function panel – Urine output	Table 6	8	8	8	Not applicable.
Serum and 24-h urine renal function panel – Serum Creatinine	Table 6	5	8	7	Exclusion of individual values identified as statistical outliers
Serum and 24-h urine renal function panel – Urine Creatinine	Table 6	6	6	6	Exclusion of individual values identified as statistical outliers
Serum and 24-h urine renal function panel – Clearance Creatinine	Table 6	6	5	7	Exclusion of individual values identified as statistical outliers
Serum and 24-h urine renal function panel – Clearance Creatinine/BW	Table 6	6	5	7	Limited by the number of values of clearance creatinine
Serum and 24-h urine renal function panel – Serum Albumin	Table 6	6	8	8	Exclusion of individual values identified as statistical outliers
Serum and 24-h urine renal function panel – Urine Albumin	Table 6	5	5	6	Exclusion of individual values identified as statistical outliers
Serum and 24-h urine renal function panel – Clearance Albumin	Table 6	5	5	6	Exclusion of individual values identified as statistical outliers
Serum and 24-h urine renal function panel – Serum Glucose	Table 6	5	8	8	Exclusion of individual values identified as statistical outliers
Serum and 24-h urine renal function panel – Urine Glucose	Table 6	6	5	8	Exclusion of individual values identified as statistical outliers
Serum and 24-h urine renal function panel – Clearance Albumin	Table 6	5	5	8	Exclusion of individual values identified as statistical outliers
Serum and 24-h urine renal function panel – Serum Sodium	Table 6	8	8	8	Not applicable.

Serum and 24-h urine renal function panel – Serum Potassium	Table 6	8	8	8	Not applicable.
Serum and 24-h urine renal function panel – Serum Phosphorous	Table 6	8	8	8	Not applicable.
WAT weight (/BW)	Table S2	8	8	8	Not applicable.
Liver weight (/BW)	Table S2	8	8	8	Not applicable.
Kidney weight (/BW)	Table S2	8	8	8	Not applicable.
Serum ALT	Table S3	8	8	7	Exclusion of individual values identified as statistical outliers
Serum AST	Table S3	8	8	8	Not applicable.

Table S2. Liver, kidney, and white adipose tissue (WAT) weights.

**Parameter**	**Chow**	**LF**	**HF**
Epididymal White Adipose Tissue (WAT) Weight (g)	7.10 ± 0.57	16.28 ± 2.64 **	17.55 ± 3.03 ***
WAT Weight/BW (g/g BW)	0.015 ± 0.001	0.030 ± 0.004 ***	0.031 ± 0.003 ***
Liver Weight (g)	16.26 ± 0.75	16.09 ± 0.99	15.82 ± 0.93
Liver Weight/BW (g/g BW)	0.034 ± 0.002	0.029 ± 0.001	0.029 ± 0.001
Kidney Weight (g)	2.93 ± 0.03	2.81 ± 0.10	2.78 ± 0.10
Kidney Weight/BW (g/g BW)	0.0061 ± 0.0002	0.0053 ± 0.0001 **	0.0049 ± 0.0002 ***

Table S3. Serum alanine aminotransferase (ALT) and aspartate aminotransferase (AST) levels.

**Parameter**	**Chow**	**LF**	**HF**
Serum ALT (U/L)	22.13 ± 0.85	23.75 ± 2.11	25.14 ± 1.22
Serum AST (U/L)	66.50 ± 3.86	70.50 ± 2.81	82.63 ± 10.75
